# The latency of spontaneous eye blinks marks relevant visual and auditory information processing

**DOI:** 10.1167/jov.21.6.7

**Published:** 2021-06-11

**Authors:** Supriya Murali, Barbara Händel

**Affiliations:** 1 Department of Psychology III, University of Würzburg, Würzburg, Germany; 2 Department of Psychology III, University of Würzburg, Würzburg, Germany

**Keywords:** spontaneous eye blink, blink latency, blink suppression, visual modality, auditory modality, temporal judgment, sensory influence, cognitive influence, attention

## Abstract

Eye blinks are influenced by external sensory and internal cognitive factors, as mainly shown in the visual domain. In previous studies, these factors corresponded to the time period of task-relevant sensory information and were often linked to a motor response. Our aim was to dissociate the influence of overall sensory input duration, task-relevant information duration, and the motor response to further understand how the temporal modulation of blinks compares among sensory modalities.

Using a visual and an auditory temporal judgment task, we found that blinks were suppressed during stimulus presentation in both domains and that the overall input length had a significant positive relationship with the length of this suppression (i.e., with the latency of the first blink after stimulus onset). Importantly, excluding the influence of the overall sensory input duration we could show that the duration of task-relevant input had an additional influence on blink latency in the visual and the auditory domain. Our findings further suggest that this influence was not based on sensory input but on top–down processes. We could exclude task difficulty and the timing of the motor response as driving factors in the blink modulation.

Our results suggest a sensory domain–independent modulation of blink latencies, introduced by changes in the length of the task-relevant, attended period. Therefore, not only do blinks mark the timing of sensory input or the preparation of the motor output, but they can also act as precise indicators of periods of cognitive processing.

## Introduction

Humans spontaneously blink about 10 to 15 times a minute (Burr, 2005; Doughty, 2001; Kaminer, Powers, Horn, Hui, & Evinger, 2011). Although a main function of these blinks is to maintain the corneal tear film, their frequency is much higher than what would be required for that purpose (Kaminer et al., 2011; Zametkin, Stevens, & Pittman, 1979). Importantly, these blinks do not occur randomly in time but show a specific modulation. Blink probability is reduced during the presentation of important sensory information and is increased after the offset of the sensory input (Bonneh, Adini, & Polat, 2016; Cong, Sharikadze, Staude, Deubel, & Wolf, 2010; Hoppe, Helfmann, & Rothkopf, 2018; Oh, Jeong, & Jeong, 2012; Siegle, Ichikawa, & Steinhauer, 2008; Wascher, Heppner, Möckel, Kobald, & Getzmann, 2015). This is also true for the auditory domain (Bauer, Strock, Goldstein, Stern, & Walrath, 1985; Kobald, Wascher, Heppner, & Getzmann, 2019; Oh, Jeong, et al., 2012). In most of these studies, the sensory input was task relevant, and the effects of task-relevant factors and the sensory offset coincided with a task-related motor response. Therefore, although the pattern of blink modulation has been well described, it is not clear if it is driven by a sensory input–induced bottom–up process or by cognitively defined task demands, or if it is linked to the motor response. We shortly review what is known about the influence of these processes on blink modulation.

Sensory input clearly affects blinking (Bonneh et al., 2016; Doughty, 2001). Bonneh et al. (2016) showed that the length of blink suppression during stimulus presentation is longer for stimuli with lower contrast and higher spatial frequency. Nevertheless, it has been suggested that the modulation of blinking is mediated by attentional mechanisms. The decrease of blinks has been discussed to reflect attention allocation and the subsequent increase to represent the point when all information processing has been completed (Wascher et al., 2015). Along the same lines, blinks have been shown to be influenced by task relevance, as well as task difficulty. The increase in blink probability following stimulus presentation is lower if no task is involved, and therefore it is not necessary for attention to be directed toward the sensory input (Brych & Händel, 2020). Accordingly, attention could be the driving factor in explaining why the suppression of blinks during sensory input is stronger for more difficult tasks (Oh, Jeong, et al., 2012). The literature clearly indicates that blinking can be modulated by top–down processes, but how much of this effect is related to the presence of sensory input itself is not known, as the duration of sensory input usually coincides with the duration of task-relevant input.

Despite sensory input–based effects, blink modulation can also happen independent from sensory input. A study by Hoppe et al. (2018) showed that a mere expectation of a stimulus could already introduce a reduction in blink probability, meaning that the sensory input itself is not necessary to drive the modulation. Such a reduction, which is due to the expectation of the input, can also be modulated by attentional factors. The decrease preceding stimulus presentation has been shown to be stronger if attention is directed toward expected visual input compared with auditory input, despite the fact that in both conditions identical audiovisual stimulation would follow (Brych & Händel, 2020). These findings indicate that blink modulation can be somewhat independent from the sensory input; however, it has not been tested how much the top–down modulation during stimulation depends on the sensory input itself.

Stimulus and task-relevant factors have not been disentangled within a single task. This is also true with regard to possible effects of the motor output. Although it has been found that tasks that do not require a motor response still lead to a modulation in blinks (Brych & Händel, 2020; Kobald et al., 2019; Wascher et al., 2015), other studies have found that blinks are in fact modulated around the motor response (Bauer et al., 1985; Oh, Jeong, et al., 2012). Additionally, blinks have been shown to be entrained by the motor response when participants engage in self-paced rhythmic finger tapping without external sensory cues (Cong et al., 2010). Considering both motor and task-related factors, Colzato, van Wouwe, and Hommel (2007) showed that blink rates reflect the strength of visuomotor binding between a task-relevant visual stimulus and a key press. The question remains if an executed task-related motor response influences blink timing.

Our goal was to understand and disassociate the influence on blinking that stems from bottom–up sensory-driven effects, the timing of the task-related response, and top–down influences, specifically focusing on the cognitively defined time of task-specific sensory information. To this end, we independently manipulated overall sensory input duration and the task-relevant sensory input duration during a comparable auditory and visual simultaneity judgment task. To secure comparable performance between the two modalities, smaller differences between the timing of the bilateral stimuli were used in the auditory task, as temporal processing has been shown to be better for the auditory domain (Kanabus, Szelag, Rojek, & Poppel, 2002). Additionally, because low-level stimulus features have been shown to affect blinking (Bonneh et al., 2016), we conducted the experiments in complete darkness. Importantly, because there is a qualitative difference between bilateral simultaneous visual and bilateral simultaneous auditory stimuli due to binaural fusion, we analyzed and discuss the influence of this specific case separately. We hypothesized that blinks mark the active processing period of task-relevant input, independent of the overall stimulation duration and independent of the timing of the planned or executed motor output, and we further predicted a comparable timing between modalities.

## Methods

Eighteen subjects (four males) between the ages of 18 and 35 years participated in the study. All participants gave their written informed consent and received either payment or study credit for their participation. The study was approved by the local ethics committee and was in line with the European general data protection regulations (DSVGO).

The mobile SMI Eye Tracking Glasses (ETG 2w Analysis Pro, 120 Hz; SensoMotoric Instruments, Teltow, Germany) were used to record eye data. The responses were given via two buttons connected to a response box (K-RB1-4; The Black Box ToolKit, Ltd, Sheffield, UK), which in turn was connected to a Dell Precision M6700 laptop (Dell Technologies, Round Rock, TX) via a USB cable. The experiment and the analysis were coded in MATLAB 2012 and 2015a (MathWorks, Natick, MA), respectively, using the Psychtoolbox extensions (Brainard, 1997; Kleiner, Brainard, & Pelli, 2007; Pelli, 1997). All data streams were recorded using the Lab Streaming Layer (LSL; https://github.com/sccn/labstreaminglayer) along with LabRecorder 1.12b. Note that the visual condition was always tested first. The experimental room was completely dark, as we used a light-tight booth used for electroencephalographic recordings with internal ventilation. The infrared light of the eye tracker did not extend into the visible range, all internal light sources were turned off or carefully wrapped in light-tight material, and no light could be detected even after staying 10 minutes inside the room.

### Visual condition

#### Participants

Of the 18 subjects, two were excluded for the visual condition because of an overall response accuracy of below 10% and a mean blink rate of below 1 blink per minute, respectively.

#### Procedure

**Figure 1.**
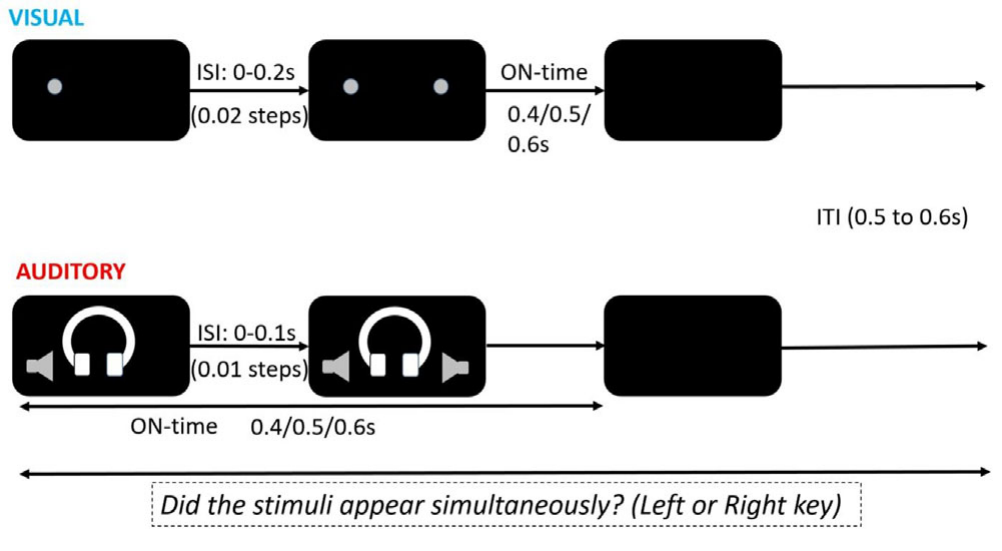
Stimulus and task during the visual and the auditory condition. The ISIs ranged from 0 to 0.2 second in the visual and 0 to 0.1 second in the auditory condition. Subjects had to judge whether or not the two stimuli appeared simultaneously. The ON-time was randomly assigned as 0.4, 0.5, or 0.6 second but was added to the ISI in the visual and not the auditory condition. Stimuli were turned off simultaneously. The intertrial interval (ITI) ranged from 0.5 to 0.6 second and was randomly assigned after the offset of the stimulus.

The visual stimulus was presented using three red light-emitting diodes (LEDs) with a diameter of 4 mm, which were placed at eye level using magnets on a horizontally mounted metal ruler at a distance of 50 cm from the participant. The central LED was only turned on at the start of the experiment and was switched off during the trials. It served to help subjects keep fixation in between the two lateral LEDs. The other two LEDs were placed on either hemifield, each at 11 degrees from the central fixation. During each trial, the two stimuli (LEDs) were turned on at the same or at different times with interstimulus intervals (ISIs) ranging from 0 to 0.2 second in steps of 0.02 second. ISI = 0 indicates that both stimuli were turned on simultaneously. In the non-simultaneous trials (ISI ≠ 0), the left stimulus appeared first in half of the trials. The order of the different trials (simultaneous and non-simultaneous, left first or right first) was randomized for each subject. After their onset, the two stimuli remained on for 0.4, 0.5, or 0.6 second, which is referred to as the stimulus ON-time, until both were turned off simultaneously. The ON-time was randomly assigned for every trial and was added to the ISIs here, but not in the auditory condition. To further add to the unpredictability of the next trial, we jittered the next stimulus onset by randomly adding an intertrial interval between 0.5 and 0.6 second after the offset of the two stimuli in the current trial (see Figure 1).

The participants had to indicate whether or not the two lights appeared simultaneously by pressing either a left or a right button (randomly assigned for each subject) with their dominant hand, as fast as possible at any time during the trial. A total of 1012 trials were presented in two sessions (11 ISIs × 46 trials × 2 sessions). To avoid excessive fatigue, given the complete darkness of the environment, we included five mandatory breaks (two during each session and one between sessions) that were taken inside the dark chamber, wherein participants were allowed to relax and close their eyes if needed.

### Auditory condition

#### Participants

Of the 18 subjects tested, one subject was excluded for a low blink rate (<1 blink per minute). For the analysis regarding ON-time and ISI, six additional subjects were excluded due to missing information. Due to a programming error, the trigger information for the ON-time and ISI was not recorded for these subjects.

#### Procedure

The auditory stimulus was presented using a PC3 Chat headset (Sennheiser, Wedemark, Germany) along with an UR22mkII external sound card (Steinberg Media Technologies, Hamburg, Germany) and consisted of two pure tones of 500 Hz, with no on/off ramps, presented to each ear. During each trial, the two stimuli turned on at the same or at different times with ISIs ranging from 0 to 0.1 second in steps of 0.01 second. Similar to the visual condition, ISI = 0 indicated that both stimuli turned on simultaneously. In the non-simultaneous trials (ISI ≠ 0), the left stimulus (presented to the left ear) appeared first in one-half of the trials. The order of the different trials (simultaneous and non-simultaneous, left ear first or right ear first) was randomized for each subject. The duration of the entire stimulation period (from onset of the first stimulus until the offset of both stimuli) was randomly assigned to the trials and lasted 0.4, 0.5, or 0.6 second. This period was termed as ON-time. Unlike in the visual condition, the ON-time was not added to the ISI. After this period, both tones were turned off simultaneously. To further add to the unpredictability of the next trial, we jittered the next stimulus onset by randomly adding an intertrial interval between 0.5 and 0.6 second after the offset of the two stimuli in the current trial (see Figure 1).

The participants had to indicate whether or not the two sounds appeared simultaneously by pressing either a left or a right button (randomly assigned for each subject) with their dominant hand, as fast as possible at any time during the trial. There were a total of 506 trials (11 ISIs × 46 trials × 1 session). To avoid excessive fatigue, we included two mandatory breaks that were taken within the dark chamber.

### Stimulus timing test

Before we began the data collection, we tested our devices to make sure that the timing was precise. This was done using a photodiode for the visual stimulus and an audio output recorder that measured the audio output directly from the audio jack. The results of this test can be found in Supplementary Figure S12.

## Analysis

### Blink detection

We computed a blink detection algorithm similar to previous studies (Brych & Händel, 2020; Brych, Murali, & Händel, 2020; Brych, Murali, & Händel, 2021), based on the pupil radius data. We first *z*-transformed the radius data. After this, a blink was detected if either data from both eyes were missing or data from one of the eyes was missing and the other eye had a *z*-value below a certain threshold (–1, –2, or –3, set individually because of considerable difference in the signal-to-noise ratios in the data). The onset and the offset of the blink were then extended until the pupil radii of either of the two eyes were higher than the set threshold. Blinks that were less than 100 ms apart were combined. Finally, blinks with durations longer than 0.5 second were discarded.

### Blink rates (time-resolved)

To visualize the modulation of blinking during a task, we plotted the mean blink modulation against the trial onset (i.e., the onset of the first stimulus) (Figure 4). We calculated the normalized mean number of blinks in each time window (0.1 second, non-overlapping) by first dividing the mean number of blinks in that time window by the mean number of blinks in all time windows for each subject. The global mean was then taken over all subjects for each time window.

### Blink latency

To test which factor influenced blink latency, we calculated (as a dependent variable) the latency of the first blink from the onset of the trial (i.e., onset of the first stimulus). The Kolmogorow–Smirnow test (KS test) revealed a non-normal distribution of blink latencies for both the visual (KS statistic = 0.64, *p* < 0.0001) and the auditory (KS statistic = 0.5506, *p* < 0.0001) condition. Therefore, a log-transformation was applied. Additionally, log-transforming all positive values has been recommended to improve the fit and predictive power of linear models (Gelman & Hill, 2006). We then conducted separate repeated-measures, two-factor analysis of covariance (ANCOVA) for the visual and auditory condition to analyze the effect of the categorical variable ON-times (0.4, 0.5, and 0.6 second) and reaction times (low and high, segregated for each subject according to the median reaction time of that subject) and the continuous predictor ISIs (0.02–0.2-second ISI for the visual and 0.01–0.1-second ISI for the auditory) on the log-transformed blink latencies.

### Reaction time

With regard to the influence on the motor output on blink latency, to test if there is a correlation on an individual level we included a linear regression between blinks latency and reaction time for each individual subject. Additionally, to understand how stimulus and task features influence the reaction time, we conducted a one-factor ANCOVA with the categorical variable ON-times (0.4, 0.5, and 0.6 second) and the continuous predictor ISIs (0.02–0.2-second ISI for the visual and 0.01–0.1-second ISI for the auditory) on the reaction time. Similar to the analysis on blink latency, we log-transformed reaction time for both the individual regressions, as well as the ANCOVA.

## Results

### Overall performance (accuracy)

**Figure 2.**
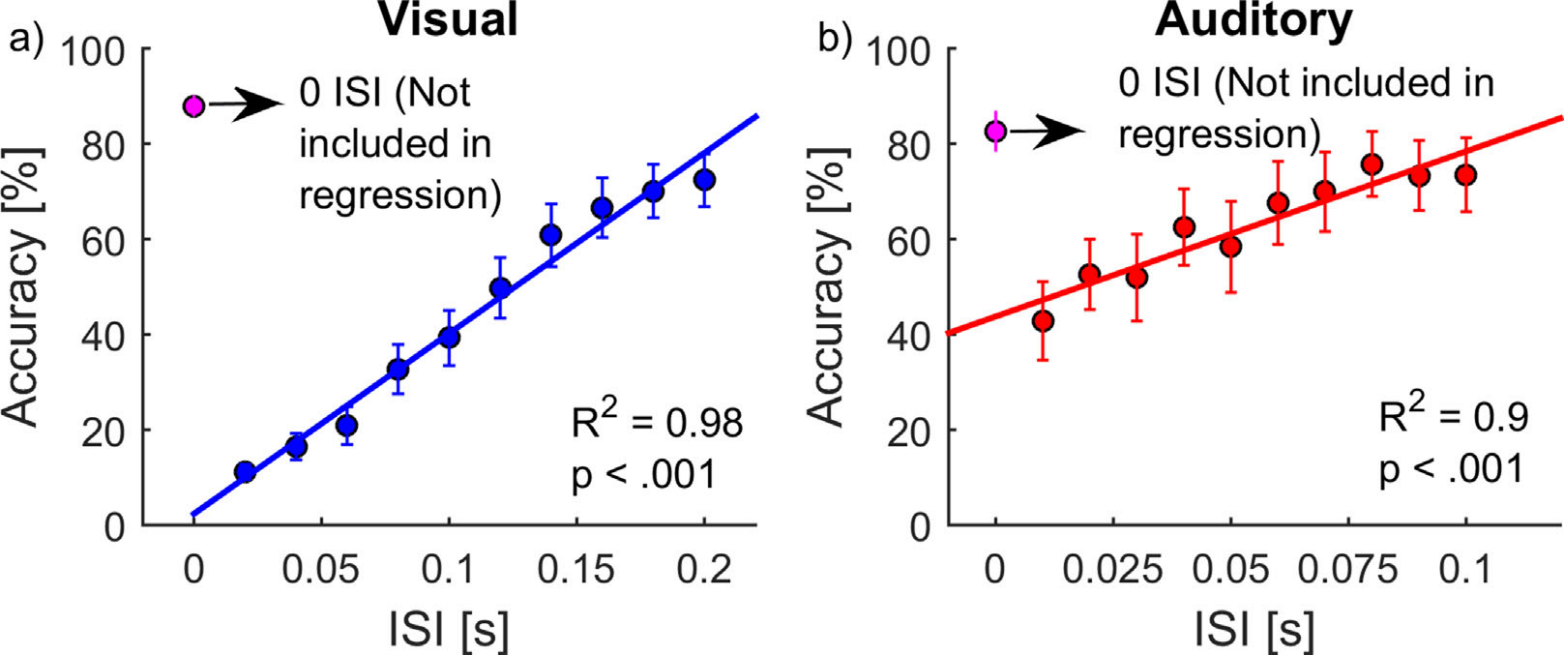
Accuracy plotted against the ISI for (a) the visual condition (*n* = 16) and (b) for the auditory condition (*n* = 11). Error bars represent standard errors.

The overall mean accuracy and reaction times for the visual condition were 48.5% (*SD* = 15.4%) and 0.56 second (*SD* = 0.22 second) respectively; for the auditory condition, they were 63.8% (*SD* = 22.5%) and 0.5 second (*SD* = 0.14 second), respectively. As predicted, for both the visual and the auditory conditions, the accuracy was high for ISI = 0. As further expected, the accuracy increased with increasing ISIs (for ISIs above 0) (Figures 2a and b). Regression analysis showed a significant linear relationship between accuracy and ISI (calculated without ISI = 0) for both the visual condition, *R^2^* = 0.98, β = 0.02, *F*(1, 8) = 361.1, *p* < 0.001, and the auditory condition, *R^2^* = 0.90, β = 0.4, *F*(1,8) = 72.4, *p* < 0.001.

### General blink parameters (overall rate and duration)

**Figure 3.**
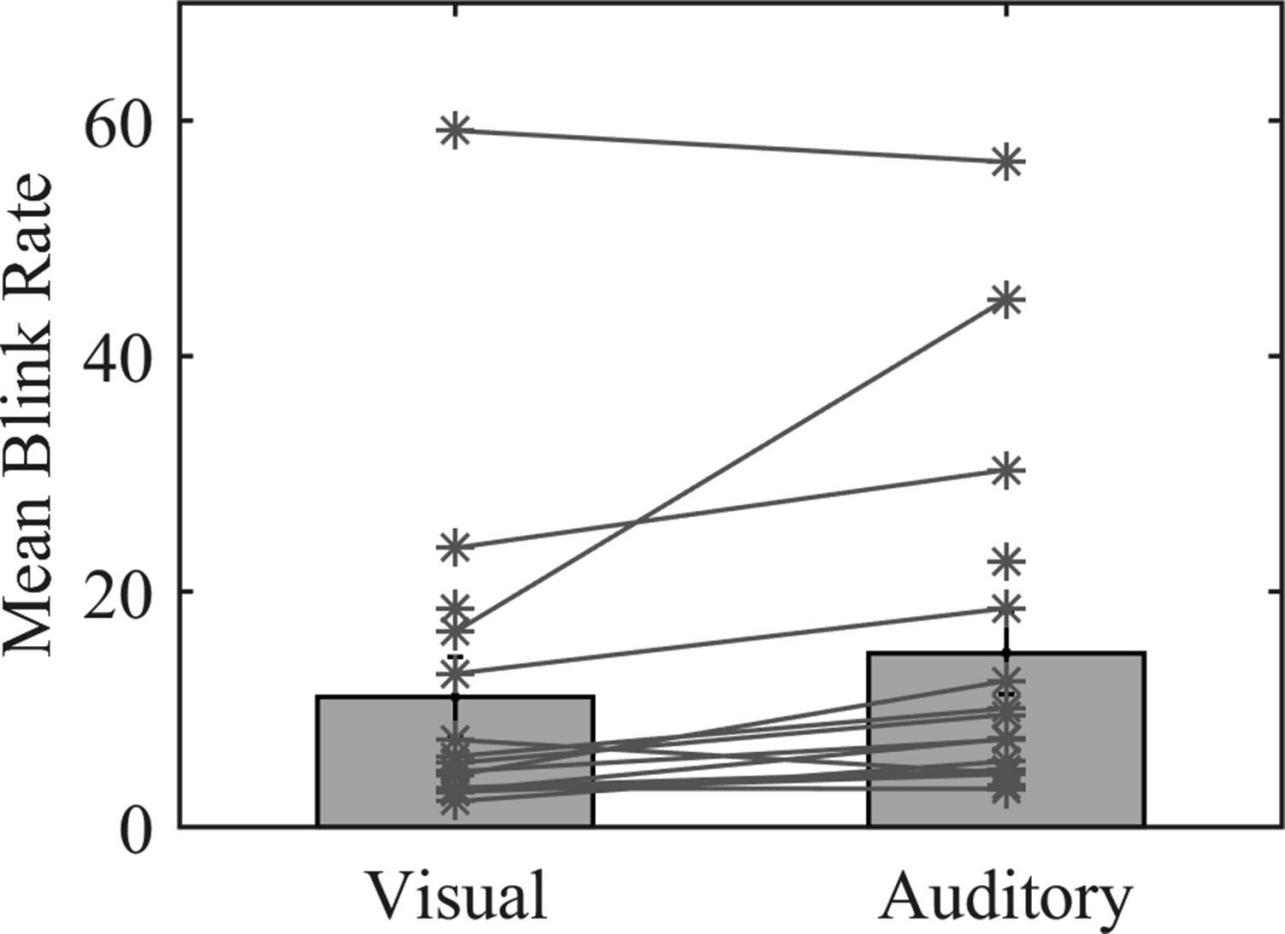
Mean blink rates (or blinks per minute) during the visual condition (*n* = 16) and the auditory condition (*n* = 17). Error bars represent standard errors. The asterisks represent data for each subject.

The mean blink rate during the visual condition was 11.05 per minute (*SE* = 3.5), and during the auditory condition it was 14.7 per minute (*SE* = 3.7), as shown in Figure 3. A *t*-test showed a significant difference between the blink rates of the domains, *t*(14) = 2.3, *p* = 0.03.

### Time resolved blink rates

**Figure 4.**
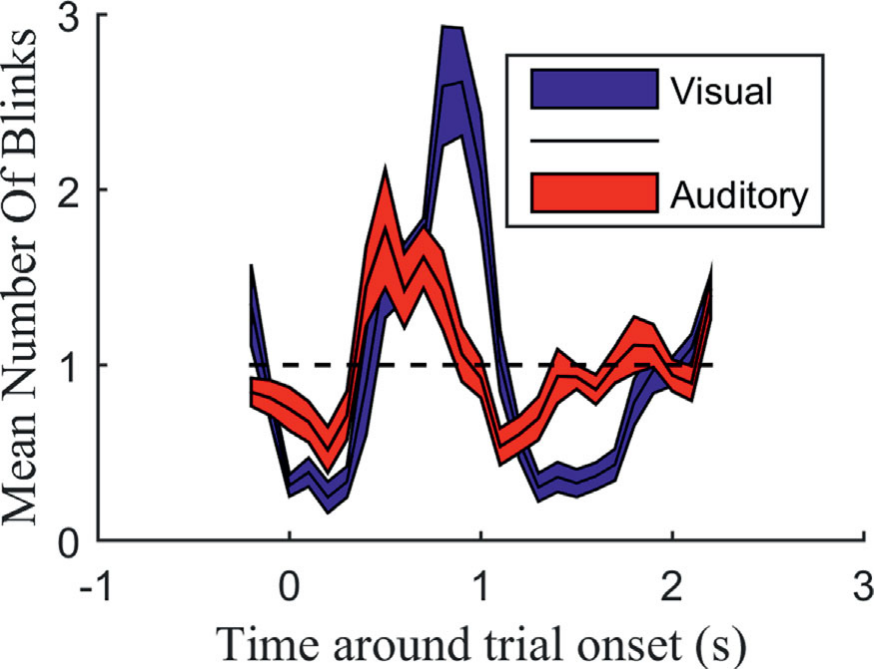
Normalized mean number of blinks during the visual and the auditory condition around trial onset (i.e., onset of the first stimulus of the pair). The *x*-axis represents timing with 0.1-second non-overlapping windows. The *y*-axis represents the normalized mean number of blinks in each window over all subjects. The normalized mean was calculated by first taking the mean number of blinks in each 0.1-second bin and dividing that by the mean number of blinks in all bins for each subject and finally taking the mean for each bin over all subjects. Therefore, a value of 1 would be the baseline number of blinks. The colored regions represent the standard error.

To visualize the modulation of blinking throughout the trial, we looked at the normalized mean number of blinks from 0.2 second before the trial onset to 2 second after the trial onset for both the modalities (Figure 4). Trial onset was defined by the onset of the first stimulus. The purpose was to see if we could replicate the previously described modulation in the visual domain and if it occurred similarly in the auditory domain. The graphs show a similar modulation in both modalities, with an increase in blinks starting at about 0.3 second and lasting until 1.2 seconds after the trial onset.

### Blink latency

To test which factors—overall stimulus (i.e., ON-time), task-relevant period (i.e., ISI), and motor output (i.e., reaction time)—have an influence on blink latency, we conducted a repeated-measures, two-factor ANCOVA for the visual and auditory modalities separately. Our results from the ANCOVA showed that there was a significant main effect of ON-time and ISI, but not for reaction time, in both modalities. Specifically, for the visual condition, there were significant effects of ON-time, *F*(2, 502) = 8.5, *p* = 0.0002, and ISI, *F*(1, 502) = 14.8, *p* = 0.0001, but not reaction time, *F*(1, 502) = 0.02, *p* = 0.88. Similarly, for the auditory, there were significant effects of ON-time, *F*(2, 357) = 3.13, *p* = 0.04, and ISI, *F*(1, 357) = 5.38, *p* = 0.02, but not reaction time, *F*(2, 357) = 5.8057e-04, *p* = 0.9. The individual influences and the corresponding post hoc tests are presented in detail below.

#### Factor ON-time

**Figure 5.**
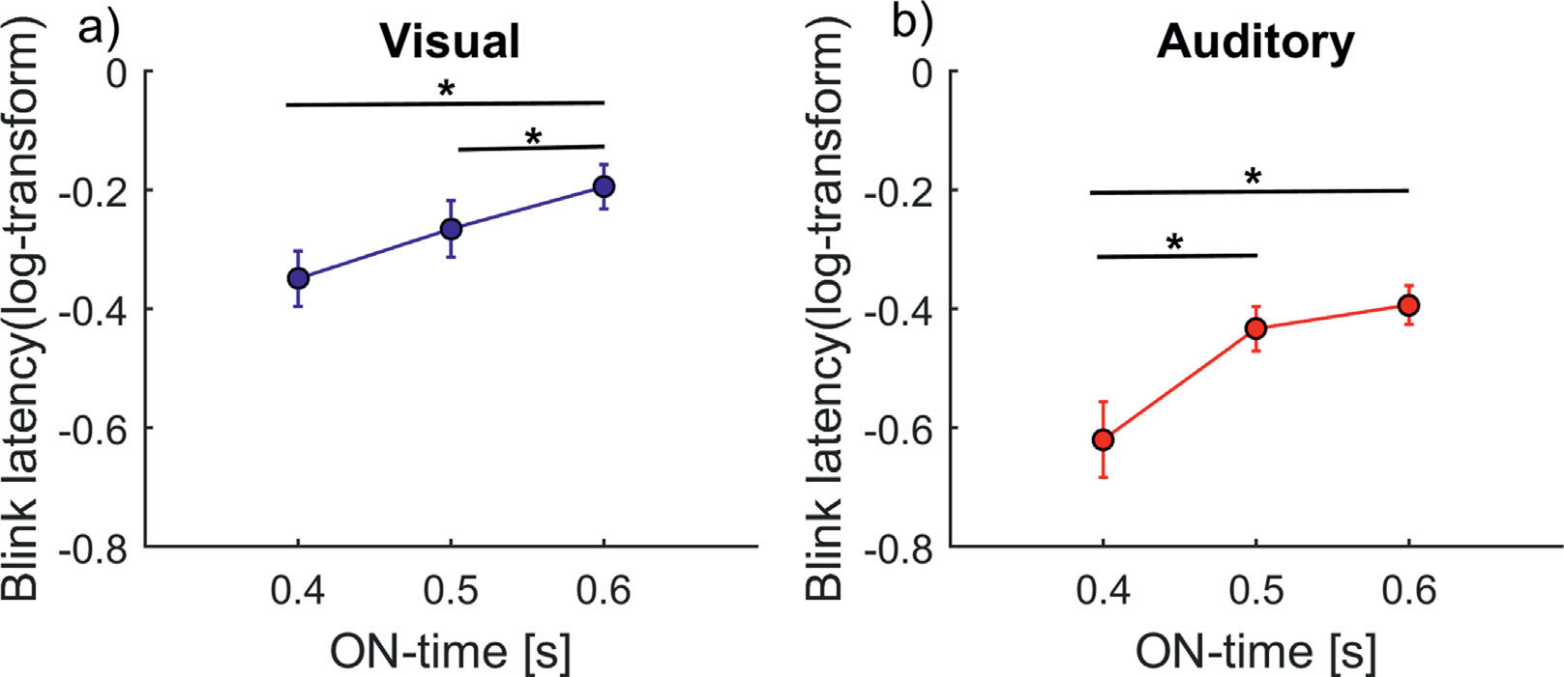
The effect of ON-time (overall stimulus duration) on blinks. Blink latency for the three ON-times in (a) the visual condition (*n* = 16) and (b) the auditory condition (*n* = 11). The *x*-axis shows the ON-times, and the *y*-axis shows the log-transformed blink latency (onset of first blink after trial onset). Error bars represent standard errors. The ANCOVA revealed a significant influence in both the visual domain, *F*(2, 502) = 8.5, *p* = 0.0002, and the auditory domain, *F*(2, 357) = 3.13, *p* = 0.04. Supplementary Figure S1 shows individual data. The asterisks indicate a significant post hoc comparison.

Figures 5a and b show the mean blink latency (log-transformed) for both the visual and auditory condition. For the visual ON-times, post hoc *t*-tests revealed significant differences between 0.4 second and 0.6 second, *t*(15) = 3.7, *p* = 0.002, and between 0.5 second and 0.6 second, *t*(15) = 3.4, *p* = 0.003, but not between 0.4 second and 0.5 second, *t*(15) = 1.9, *p* = 0.07. For the auditory condition, there were significant differences between 0.4 second and 0.5 second, *t*(10) = 3.4, *p* = 0.007, and between 0.4 second and 0.6 second, *t*(10) = 2.7, *p* = 0.02, but not between 0.5 second and 0.6 second, *t*(10) = 0.74, *p* = 0.5 (see Supplementary Figure S1 for individual data). Additionally, since the ISI added to the ON-time during the visual condition, Supplementary Figure S9 shows that the increase in blink latency with increasing ON-time was observable for most ISIs. Therefore, the influence was not due to the time added by the ISI in the visual condition.

#### Factor ISI

**Figure 6.**
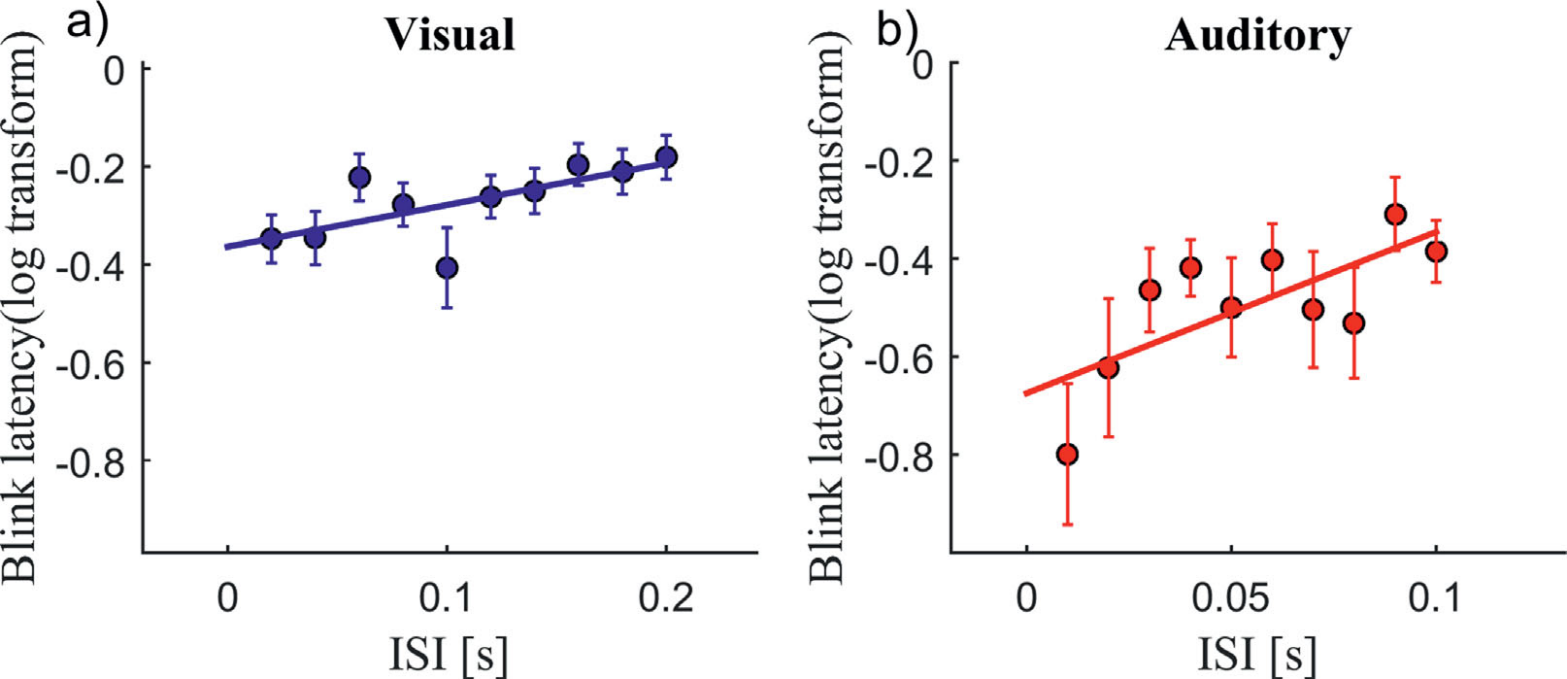
The effect of ISI (task-relevant input) on blinks. Blink latency plotted against the ISI in (a) the visual condition (*n* = 16) and (b) the auditory condition (*n* = 11). The *x*-axis shows the ISIs, and the *y*-axis shows the log-transformed blink latency (onset of first blink after trial onset). Error bars represent standard errors. The ANCOVA revealed a significant effect in both the visual domain, *F*(1, 502) = 14.8, *p* = 0.0001, and the auditory domain, *F*(1, 357) = 5.38, *p* = 0.02. Supplementary Figure S2 shows individual data.

Figures 6a and b show blink latency plotted against ISIs for both the visual and auditory modalities. In the supplementary material, we show this relationship for each ON-time (Supplementary Figure S7) and each reaction time (Supplementary Figure S8). Based on the results from the ANCOVA, it is clear that blink latencies increase with increasing ISIs. Note that, all subjects did not contribute to all ISIs in the auditory condition, because no blink was executed for some of the trials; therefore, no latency could be calculated (see Supplementary Figure S2 for individual data).

**Figure 7.**
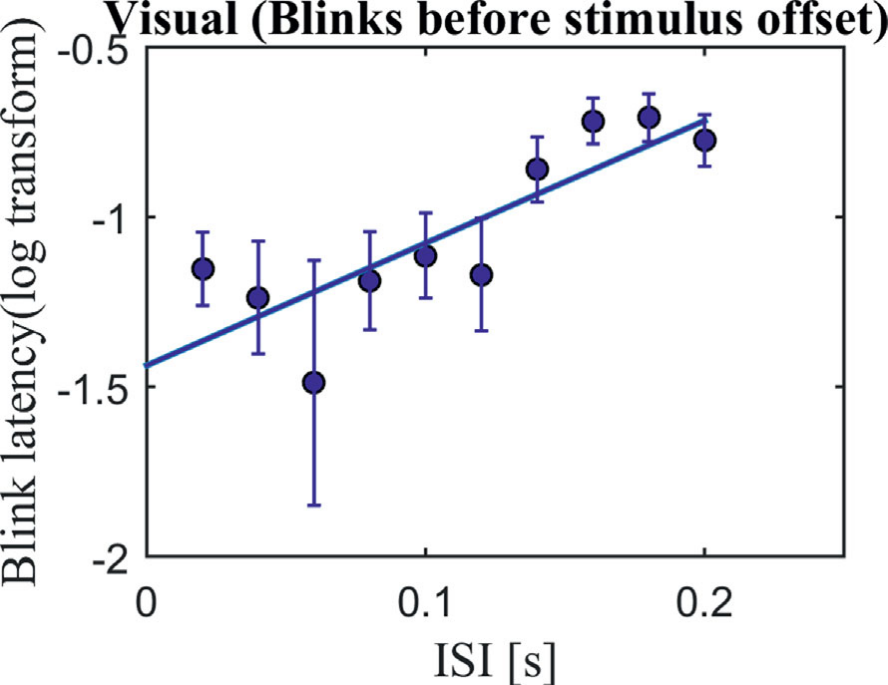
The effect of ISI (task-relevant input) on blinks occurring before the offset. Blink latency is plotted against the ISI in the visual condition (*n* = 16). The *x*-axis shows the ISIs, and the *y*-axis shows the log-transformed blink latency (onset of first blink after trial onset but only if this blink occurred before stimulus offset). Error bars represent standard errors. A linear regression model showed a significant effect, *F*(1, 125) = 12.1, *r*^2^ = 0.2, *p* = 0.0005. Supplementary Figure S3 shows individual data.

Because the ISI in the visual task was added to the ON-time (i.e., both factors defined the total stimulus duration), we wanted to see if the effect of the ISI on the blink latency was indeed due to increasing task-relevant information processing or merely due to its effect on the timing of the offset. Hence, we took only those trials wherein the first blink occurred before the end of the ON-time in the visual task (Figure 7). A linear regression model still showed a significant effect of ISI on blink latency, *F*(1, 125) = 12.1, *r*^2^ = 0.2, *p* = 0.0005. Note that, because only blinks before offset were taken into account, the number of usable data points is reduced, as not all subjects contributed to all ISIs; for some subjects, no blink was executed at all and therefore no latency could be calculated (see Supplementary Figure S3).

**Figure 8:**
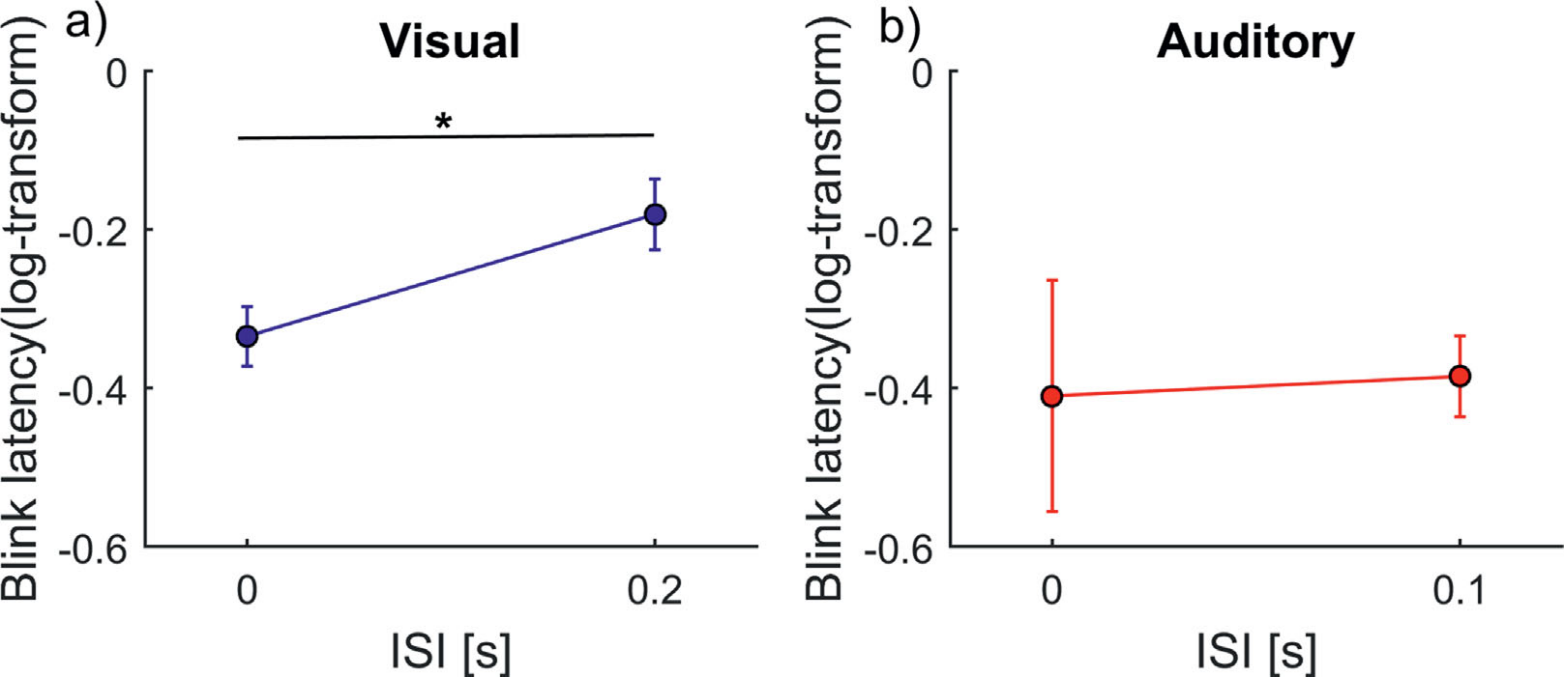
*.* Blink latency plotted against the 0 versus highest ISIs in (a) the visual condition (*n* = 16) and (b) the auditory condition (*n* = 11). The *x*-axis shows the ISIs, and the *y*-axis shows the log-transformed blink latency (onset of first blink after trial onset). Error bars represent standard errors. The *t*-tests revealed a significant difference in the visual condition, *t*(15) = 5.8, *p* = 3.5989e-05, but not in the auditory condition, *t*(10) = 0.14, *p* = 0.9. Supplementary Figure S4 shows individual data.

As noted earlier, ISI = 0 was excluded from the ANCOVA and compared separately to the highest ISI. Note that it breaks from the pattern observed in Figure 8 in the auditory condition but not in the visual condition. A *t*-test showed that there was a significant difference between ISIs of 0 second and 0.2 second in the visual condition, *t*(15) = 5.8, *p* = 3.5989e-05, but not in the auditory condition, *t*(10) = 0.14, *p* = 0.9.

**Figure 9.**
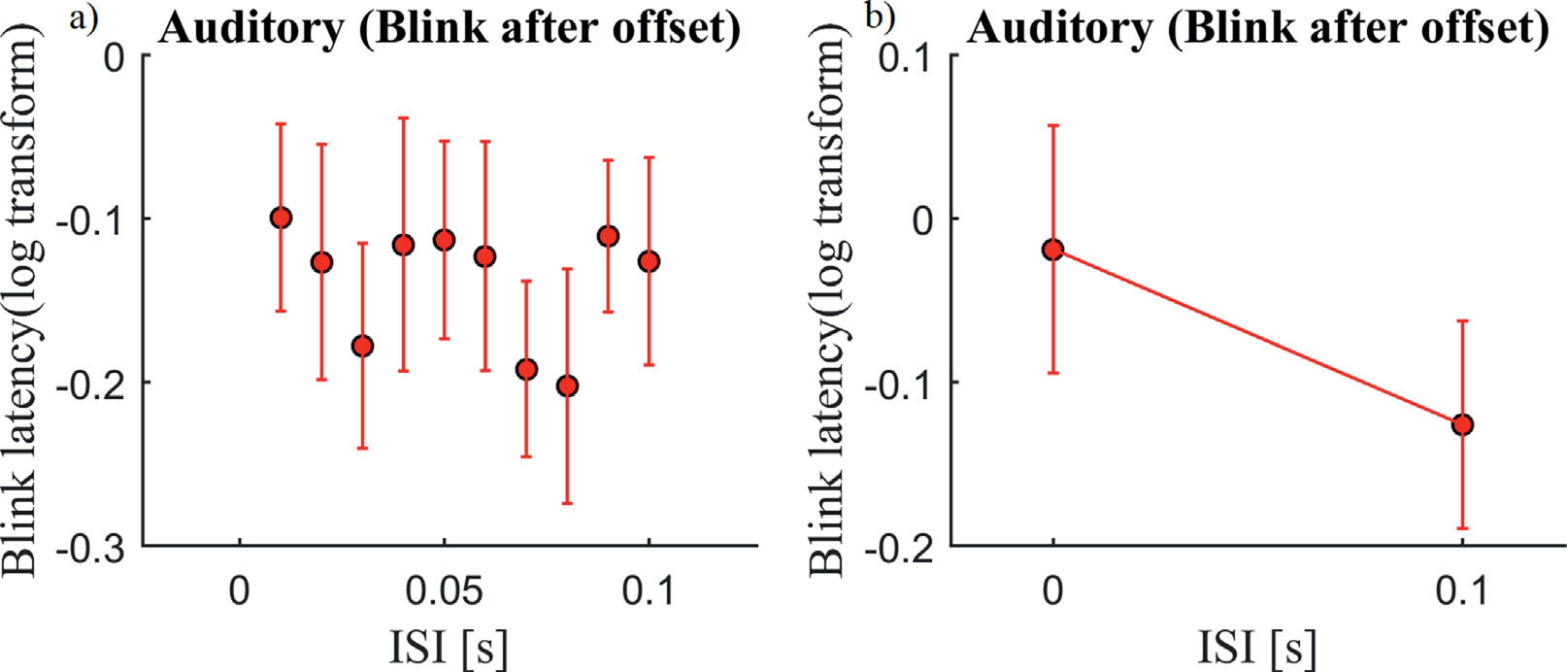
Blink latency plotted against the ISIs (a) and for the 0 and 0.1-second ISIs (b) in the auditory condition (*n* = 10; one subject had no blinks after stimulus offset for ISI = 0). Only those blinks that occurred after stimulus offset were included. The *x*-axis shows the ISIs, and the *y*-axis shows the log-transformed blink latency (onset of first blink after trial onset but only if this blink occurred after stimulus offset). Error bars represent standard errors. A two-factor ANCOVA (excluding ISI = 0) revealed no significant effect of ISI, *F*(1, 305) = 0.9, *p* = 0.3. A *t*-test between the 0-second and the 0.1-second ISIs revealed a *p* value of 0.05, *t*(9) = 2.2. Supplementary Figure S5 shows individual data.

To see if the blink latency in the auditory domain was driven by the offset of the stimulus, we exclusively looked at the blinks that occurred after stimulus offset (Figure 9) and conducted a two-factor ANCOVA (excluding ISI = 0), which revealed no significant effect of ISI, *F*(1, 305) = 0.9, *p* = 0.3. Therefore, we concluded that the ISI had no influence on the blink latency. However, ISI = 0 had a higher latency than the rest. A *t*-test between the 0-second and 0.1-second ISIs revealed a *p* value of 0.05, *t*(9) = 2.2. Note that we specifically looked at the auditory condition here because the visual ISIs added to the offset times. Because only blinks after stimulus offset were taken into account, the number of usable data points was reduced because not all subjects contributed to all ISIs (see Supplementary Figure S5 for the individual data).

#### Factor RT

**Figure 10.**
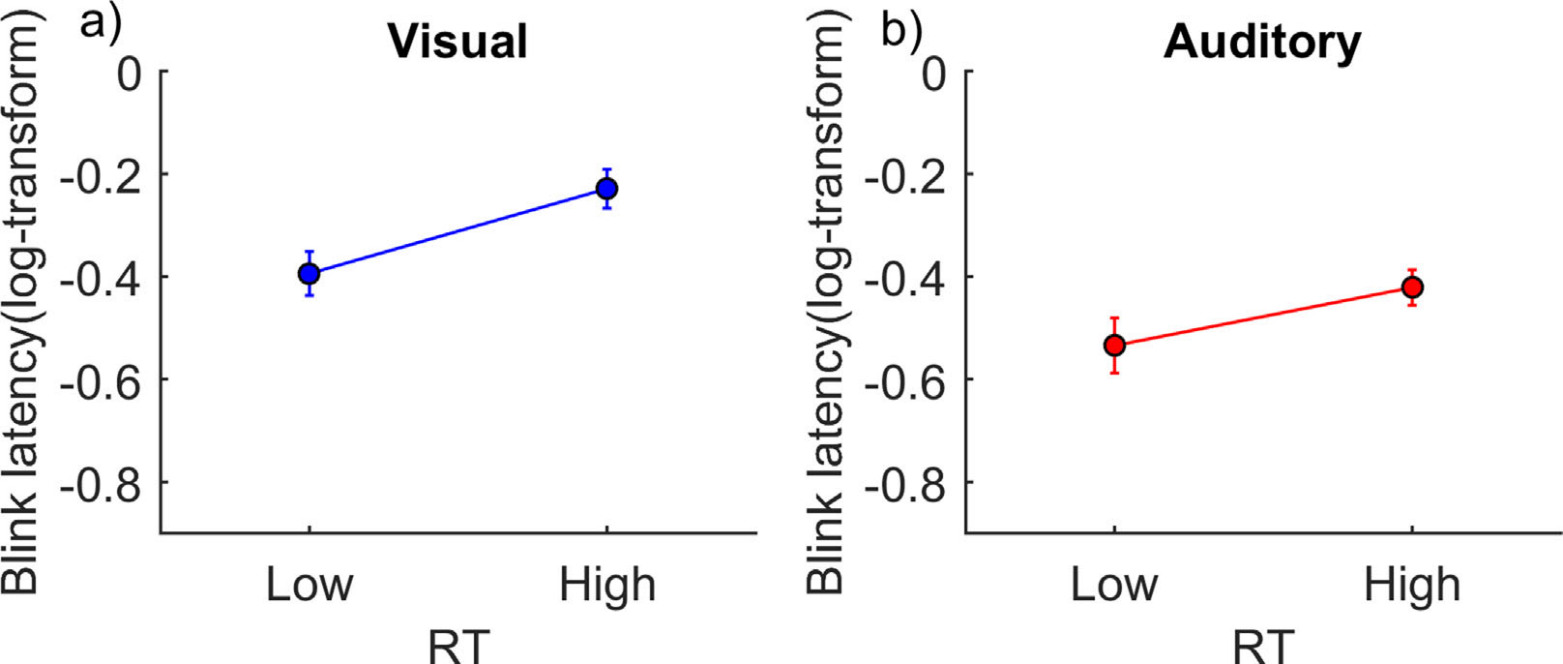
The effect of reaction time. Blink latency for the low and high reaction times in (a) the visual condition (*n* = 16) and (b) the auditory condition (*n* = 11). The *x*-axis shows reaction times, and the *y*-axis shows the log-transformed blink latency (onset of first blink after trial onset). Error bars represent standard errors. The reaction times were segregated for each subject based on the median reaction time of all trials of that subject. Note that the ANCOVA showed no effect in either the visual domain, *F*(1, 502) = 0.02, *p* = 0.88, or the auditory domain, *F*(2, 357) = 5.8057e-04, *p* = 0.9. Supplementary Figure S6 shows individual data.

**Figure 11.**
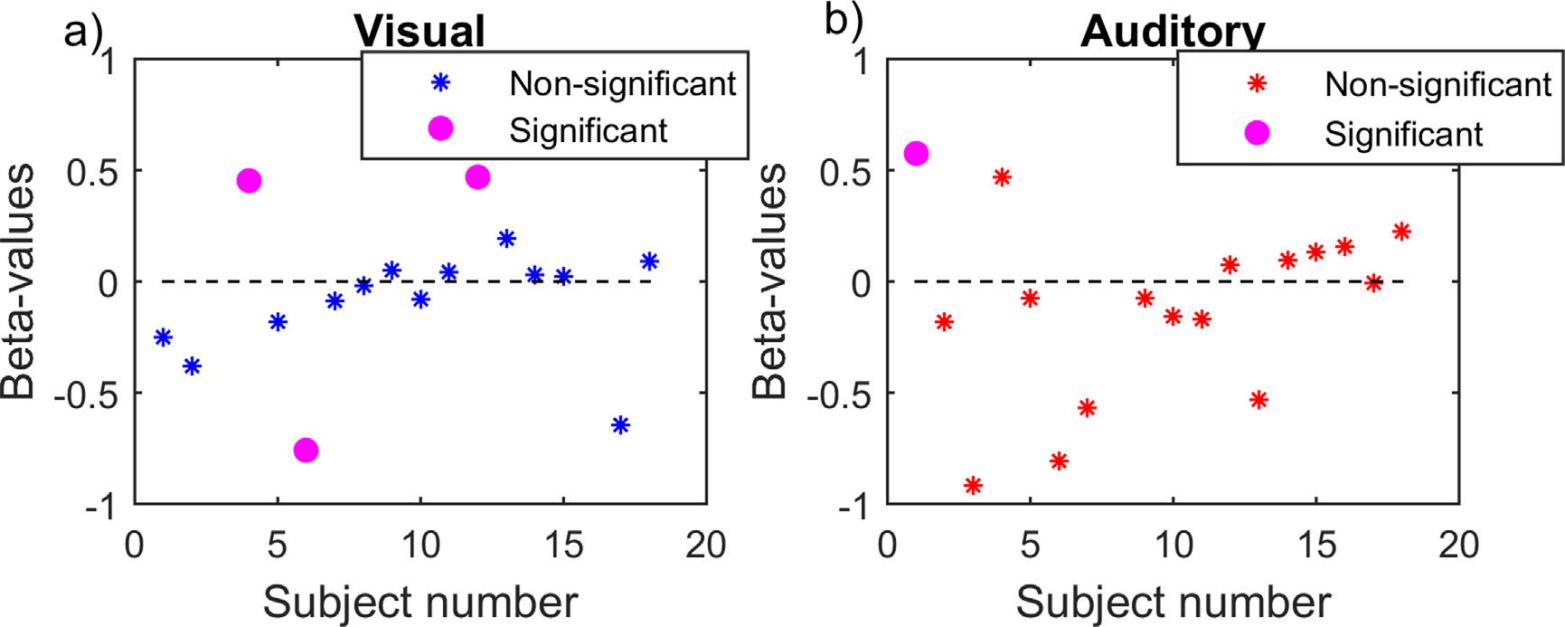
β values from linear regressions conducted on blink latency versus reaction time for each subject separately (*n* = 16 for visual and *n* = 17 for auditory). Asterisks represent subjects that showed a *p* value above 0.05 for the regression analysis, and circles represent subjects that showed a *p* value below 0.05.

Figure 10 shows the blink latency (log-transformed) for the trials with high and low reaction times. Reaction times were divided for each subject based on the median reaction time of all trials for that specific subject. The ANCOVA showed no significant influence in either domain. Although the reaction time (as divided in two categories, low and high) did not have a significant effect on the blink latency, there could still be a correlation on an individual level. We, therefore, conducted a two-sided linear regression between blink latency and reaction time for each individual subject. Figure 11 shows the β values from the regression for each subjects, with stars representing subjects that showed a *p* value above 0.05 for the regression analysis and circles representing those that showed a *p* value below 0.05. See Supplementary Tables S1 and S2 for the β values, *r*^2^ values, *F* values, and *p* values for each individual subject for the visual and auditory conditions. While Figure 11 and Supplementary Tables S1 and S2 indicated no relationship between blink latency and RT, Supplementary Figure S10 and S11 showed that the offset of sensory input influences RT.

## Discussion

Using an auditory and a visual simultaneity judgment task, our study shows a temporally precise modulation of blink latency, influenced by sensory and cognitive factors in both the auditory and visual domain. Specifically, periods of stimulus presentation were associated with a low blink rate, followed by an increase after the offset. Our aim was to understand what influences this modulation by investigating the duration of this blink suppression. To this end, we specifically looked at the influence of the following factors on the blink latency: the duration of the overall sensory input, the time of the manual response (reaction time), the duration of the task specific sensory input (ISI), and (indirectly) task difficulty. An understanding of whether these factors have similar influences across modalities, using comparable tasks, is missing to date.

The overall sensory input duration showed a robust effect, as longer stimulus ON-times led to increased blink latency (Figure 5). A modulation of blinking due to sensory input has been reported before in both the visual domain (Oh, Jeong, et al., 2012; Siegle et al., 2008) and the auditory domain (Oh, Han, Peterson, & Jeong, 2012; Oh, Jeong, et al., 2012). The low blink rate during sensory input has been interpreted as an active suppression, which is then followed by a rebound (Bonneh et al., 2016; Hoppe et al., 2018; Oh, Jeong, et al., 2012). Our results further strengthen the domain generality of this sensory-induced effect on blinking.

Importantly, our results show that the influence on blink latency goes beyond just the sensory input duration. We found that task-specific sensory input (ISI) was significantly associated with increasing blink latencies independent of the overall sensory input duration (Figure 6b, auditory; Figure 7, visual). There are several possibilities that might underlie the influence of the ISI, including change in sensory input, task difficulty, and duration of task-relevant input.

Any change of sensory input might modulate blinking. In our task, there were three changes in sensory input: the onset of stimulus one, the onset of stimulus two, and the offset of both stimuli. We discussed earlier that the offset modulated blink latency. Further, because the onset of the first stimulus was identical for all ISIs, it cannot explain the influence of ISI. We therefore can conclude that it was specifically the second stimulus that shaped the mean latency of the first blink. However, data from the auditory domain suggest that the blink latency is not driven by the second bottom–up sensory onset time that is defined by the ISI. For visual stimuli, two spatially non-overlapping but temporally overlapping signals can be perceived easily as two separate inputs. However, when two auditory signals are sufficiently temporally correlated between the ears they are fused and interpreted as a single auditory event. This is referred to as binaural fusion (Blauert, 1938; Broadbent, 1955; Leakey, Sayers, & Cherry, 1958). ISI = 0 in the auditory domain, therefore, has a separate left and right ear input but results in a single perceived onset. If it were indeed the sensory input driving the blink latency for ISI = 0, we would expect a short blink latency after visual and auditory stimulus onset. However, the data suggest an increased blink latency for the ISI = 0 condition in the auditory domain (Figure 9), indicating that the influence of ISI is not based on a bottom–up effect of sensory offset but rather on the perception of the first and second stimulus in order to solve the task.

The specific case of ISI = 0 further helps us to exclude effects related to task difficulty. Please note that, in order to influence the blink latency within an ongoing trial, the difficulty must be perceived during this period. In our paradigm, the accuracy did not linearly correlate with the perception of task difficulty. For example, for short ISIs, the probability of indicating a simultaneous appearance was way above chance level, so subjects mostly perceived it wrongly. However, because they did not receive feedback, they likely thought their responses were correct and therefore did not consider the task to be difficult. Additionally, as no feedback was provided, the perceived task difficulty could not be adapted; therefore, it is response probability rather than accuracy that marks the perceived task difficulty. The auditory ISI = 0 has a task difficulty similar to that for visual ISI = 0 (namely, above 80% response probability for simultaneous judgment). However, as the blink latency behaves differently for auditory ISI = 0 and visual ISI = 0, we conclude that task difficulty cannot be the driving factor. Additionally, the previously described influence of task difficulty (Drew, 1951; Goldstein, Bauer, & Stern, 1992; Veltman & Gaillard, 1998) always showed a positive correlation with blinking; that is, the more difficult the task, the later the blink. The visual ISI = 0 condition clearly shows a different trend.

As we have reasoned above, our findings suggest that it is neither task difficulty-related effects nor bottom–up sensory onset effects that underlie the ISI driven modulation of blink latency. However, our data are in line with the interpretation that the duration of task-relevant input influences blink latency. Although in the visual domain the task-relevant input duration increased with ISI, this was also true for the auditory domain, except for ISI = 0. Synchronous auditory inputs would usually be fused, and only after the assurance that no second stimulus was presented could the subject conclude that the first input must have consisted of two stimuli. This increases the processing time required for auditory simultaneous input. An influence of the duration of task-relevant input therefore can explain the observed pattern of blink latency, as well as the prolonged latency following ISI = 0 in the auditory domain. Of course, the timing needed to process the task-relevant information is closely related to attentional allocation toward this processing. Indeed, it has been argued that the suppression of blinks is associated with increased attention toward sensory input, and the subsequent increase in blinks has been argued to mark the end of the attentional period— that is, when all information processing is completed (Kobald et al., 2019; Wascher et al., 2015). Our findings strengthen such interpretation by showing effects that are independent of the overall sensory input duration. Additionally, our data show that it is a domain-general top–down effect on blink suppression.

Finally, our results showed that reaction time is largely independent from blink latency. As seen in Figure 11, only three out of 16 subjects in the visual condition and one out of 17 subjects in the auditory condition showed a significant relationship between reaction time and blink latency. Interestingly, some showed a negative relationship and others showed a positive one. This suggests that individual differences drive the relationship between blinks and motor responses. Studies have argued for a link between blinks and button presses because they involve overlapping brain regions (Cong et al., 2010) and activate overlapping medial frontal structures (Hanakawa, Dimyan, & Hallett, 2008). Additionally, the supplementary motor area has been shown to be involved in different endogenous motor actions (Halsband, Ito, Tanji, & Freund, 1993), as well as endogenous blinks (Jenkins, Jahanshahi, Jueptner, Passingham, & Brooks, 2000). Hence, individual differences in the amount of activation and overlap between these different cortical areas might mediate the relationship between blinks and motor responses. Another relevant individual difference might be related to dopamine. Striatal dopamine level has been shown to be positively correlated with the blink rate (Karson, 1983; Taylor, Elsworth, Lawrence, Sladek, Roth, & Redmond, 1999), whereas it shows a negative correlation with reaction time (Pullman, Watts, Juncos, Chase, & Sanes, 1988; Rihet, Possamaï, Micallef-Roll, Blin, & Hasbroucq, 2002). An individually high or low dopamine level might therefore boost a specific relationship between blinking and other motor responses. Future studies are needed to identify the factors that lead to individual occurrences of a co-modulation between different motor outputs.

In summary, although a decrease in blinks was mainly associated with sensory input, our results show that minute changes of task-relevant information length, independent of ongoing sensory stimulation, modulate blink behavior in the auditory domain and the visual domain. Our study, therefore, highlights domain-general top–down influences that can precisely modulate the timing of blinking, mapping small temporal changes in sensory-attentional demands.

## Supplementary Material

Supplement 1

Supplement 2

Supplement 3

Supplement 4

Supplement 5

Supplement 6

Supplement 7

Supplement 8

Supplement 9

Supplement 10

Supplement 11

Supplement 12

Supplement 13

## References

[bib1] Bauer, L. O., Strock, B. D., Goldstein, R., Stern, J. A., & Walrath, L. C. (1985). Auditory discrimination and the eyeblink. *Psychophysiology,* 22(6), 636–641. 4089089 10.1111/j.1469-8986.1985.tb01660.x

[bib2] Blauert, J. (1938). Review paper: Psychoacoustic binaural phenomena. In: R. Klinke & R. Hartmann (Eds.), *HEARING—Physiological bases and psychophysics* (pp. 182–189). Berlin: Springer.

[bib3] Bonneh, Y. S., Adini, Y., & Polat, U. (2016). Contrast sensitivity revealed by spontaneous eyeblinks: Evidence for a common mechanism of oculomotor inhibition. *Journal of Vision,* 16(7):1, 1–15, 10.1167/16.7.1. 27135194

[bib4] Brainard, D. H. (1997). The Psychophysics Toolbox. *Spatial Vision,* 10(4), 433–436. 9176952

[bib5] Broadbent, D. (1955). A note on binaural fusion. *Quarterly Journal of Experimental Psychology,* 7(1), 46–47.

[bib6] Brych, M., & Händel, B. (2020). Disentangling top-down and bottom-up influences on blinks in the visual and auditory domain. *International Journal of Psychophysiology,* 158, 400–410. 33181189 10.1016/j.ijpsycho.2020.11.002

[bib7] Brych, M., Murali, S., & Händel, B. (2020). How the motor aspect of speaking influences the blink rate. Retrieved from https://www.biorxiv.org/content/10.1101/2020.07.31.230391v1.full.pdf. 10.1371/journal.pone.0258322 PMC8500445 34624051

[bib8] Brych, M., Murali, S., & Händel, B. (2021). The role of blinks, microsaccades and their retinal consequences in bistable motion perception. *Frontiers in Psychology,* 12, 647256. 33897552 10.3389/fpsyg.2021.647256 PMC8061730

[bib9] Burr, D. (2005). Vision: In the blink of an eye. *Current Biology,* 15(14), R554–R556. 16051164 10.1016/j.cub.2005.07.007

[bib10] Colzato, L. S., van Wouwe, N. C., & Hommel, B. (2007). Spontaneous eyeblink rate predicts the strength of visuomotor binding. *Neuropsychologia,* 45(10), 2387–2392. 17433381 10.1016/j.neuropsychologia.2007.03.004

[bib11] Cong, D.-K., Sharikadze, M., Staude, G., Deubel, H., & Wolf, W. (2010). Spontaneous eye blinks are entrained by finger tapping. *Human Movement Science,* 29(1), 1–18. 19913931 10.1016/j.humov.2009.08.003

[bib12] Doughty, M. J. (2001). Consideration of three types of spontaneous eyeblink activity in normal humans: during reading and video display terminal use, in primary gaze, and while in conversation. *Optometry and Vision Science,* 78(10), 712–725. 11700965 10.1097/00006324-200110000-00011

[bib13] Drew, G. C. (1951). Variations in reflex blink-rate during visual-motor tasks. *Quarterly Journal of Experimental Psychology,* 3(2), 73–88.

[bib14] Gelman, A., & Hill, J. (2006). *Data analysis using regression and multilevel/hierarchical models*. Cambridge, UK: Cambridge University Press.

[bib15] Goldstein, R., Bauer, L. O., & Stern, J. A. (1992). Effect of task difficulty and interstimulus interval on blink parameters. *International Journal of Psychophysiology,* 13(2), 111–117. 1399750 10.1016/0167-8760(92)90050-l

[bib16] Halsband, U., Ito, N., Tanji, J., & Freund, H.-J. (1993). The role of premotor cortex and the supplementary motor area in the temporal control of movement in man. *Brain,* 116(1), 243–266. 8453461 10.1093/brain/116.1.243

[bib17] Hanakawa, T., Dimyan, M. A., & Hallett, M. (2008). The representation of blinking movement in cingulate motor areas: A functional magnetic resonance imaging study. *Cerebral Cortex,* 18(4), 930–937. 17652462 10.1093/cercor/bhm129

[bib18] Hoppe, D., Helfmann, S., & Rothkopf, C. A. (2018). Humans quickly learn to blink strategically in response to environmental task demands. *Proceedings of the National Academy of Sciences, USA,* 115(9), 2246–2251. 10.1073/pnas.1714220115 PMC5834680 29444860

[bib19] Jenkins, I. H., Jahanshahi, M., Jueptner, M., Passingham, R. E., & Brooks, D. J. (2000). Self-initiated versus externally triggered movements: II. The effect of movement predictability on regional cerebral blood flow. *Brain,* 123(6), 1216–1228. 10825359 10.1093/brain/123.6.1216

[bib20] Kaminer, J., Powers, A. S., Horn, K. G., Hui, C., & Evinger, C. (2011). Characterizing the spontaneous blink generator: an animal model. *Journal of Neuroscience,* 31(31), 11256–11267. 21813686 10.1523/JNEUROSCI.6218-10.2011 PMC3156585

[bib21] Kanabus, M., Szelag, E., Rojek, E., & Poppel, E. (2002). Temporal order judgement for auditory and visual stimuli. *Acta Neurobiologiae Experimentalis,* 62(4), 263–270. 12659292 10.55782/ane-2002-1443

[bib22] Karson, C. N. (1983). Spontaneous eye-blink rates and dopaminergic systems. *Brain,* 106(3), 643–653. 6640274 10.1093/brain/106.3.643

[bib23] Kleiner, M., Brainard, D., & Pelli, D. (2007). What's new in Psychtoolbox-3? *Perception,* 36(14), 1–16.

[bib24] Kobald, S. O., Wascher, E., Heppner, H., & Getzmann, S. (2019). Eye blinks are related to auditory information processing: evidence from a complex speech perception task. *Psychological Research,* 83(6), 1281–1291. 29353461 10.1007/s00426-017-0952-9

[bib25] Leakey, D., Sayers, B. M., & Cherry, C. (1958). Binaural fusion of low-and high-frequency sounds. *The Journal of the Acoustical Society of America,* 30(3), 222.

[bib26] Oh, J., Han, M., Peterson, B. S., & Jeong, J. (2012). Spontaneous eyeblinks are correlated with responses during the Stroop task. *PLoS One,* 7(4), e34871. 22493720 10.1371/journal.pone.0034871 PMC3321041

[bib27] Oh, J., Jeong, S.-Y., & Jeong, J. (2012). The timing and temporal patterns of eye blinking are dynamically modulated by attention. *Human Movement Science,* 31(6), 1353–1365. 22877514 10.1016/j.humov.2012.06.003

[bib28] Pelli, D. G. (1997). The VideoToolbox software for visual psychophysics: Transforming numbers into movies. *Spatial Vision,* 10(4), 437–442. 9176953

[bib29] Pullman, S., Watts, R., Juncos, J., Chase, T., & Sanes, J. (1988). Dopaminergic effects on simple and choice reaction time performance in Parkinson's disease. *Neurology,* 38(2), 249. 3340288 10.1212/wnl.38.2.249

[bib30] Rihet, P., Possamaï, C.-A., Micallef-Roll, J., Blin, O., & Hasbroucq, T. (2002). Dopamine and human information processing: a reaction-time analysis of the effect of levodopa in healthy subjects. *Psychopharmacology,* 163(1), 62–67. 12185401 10.1007/s00213-002-1127-x

[bib31] Siegle, G. J., Ichikawa, N., & Steinhauer, S. (2008). Blink before and after you think: Blinks occur prior to and following cognitive load indexed by pupillary responses. *Psychophysiology,* 45(5), 679–687. 18665867 10.1111/j.1469-8986.2008.00681.x

[bib32] Taylor, J., Elsworth, J., Lawrence, M., Sladek, J. Jr., Roth, R., & Redmond, D. Jr. (1999). Spontaneous blink rates correlate with dopamine levels in the caudate nucleus of MPTP-treated monkeys. *Experimental Neurology,* 158(1), 214–220. 10448434 10.1006/exnr.1999.7093

[bib33] Veltman, J., & Gaillard, A. (1998). Physiological workload reactions to increasing levels of task difficulty. *Ergonomics,* 41(5), 656–669. 9613226 10.1080/001401398186829

[bib34] Wascher, E., Heppner, H., Möckel, T., Kobald, S. O., & Getzmann, S. (2015). Eye-blinks in choice response tasks uncover hidden aspects of information processing. *EXCLI Journal,* 14, 1207–1218. 27152110 10.17179/excli2015-696 PMC4849103

[bib35] Zametkin, A. J., Stevens, J. R., & Pittman, R. (1979). Ontogeny of spontaneous blinking and of habituation of the blink reflex. *Annals of Neurology,* 5(5), 453–457. 223495 10.1002/ana.410050509

