# Supplementary figures and images for: The latency of spontaneous eye blinks marks relevant visual and auditory information processing
Source: J Vis. 2021 Jun 11;21(6):7. doi: 10.1167/jov.21.6.7 (PMC8196427; doi:10.1167/jov.21.6.7)

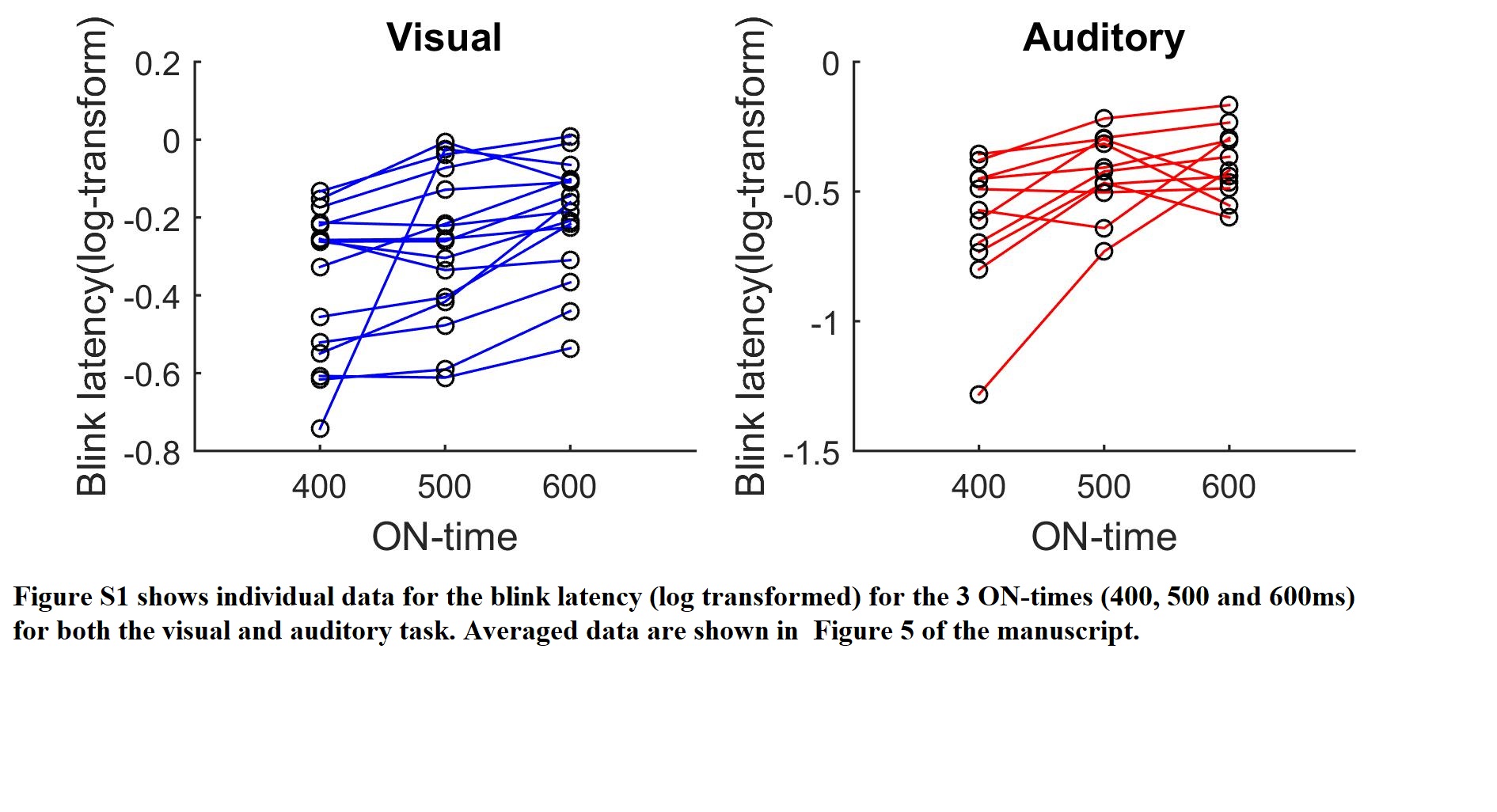

Supplement: Supplement 1 [file jovi-21-6-7_s001.jpeg]

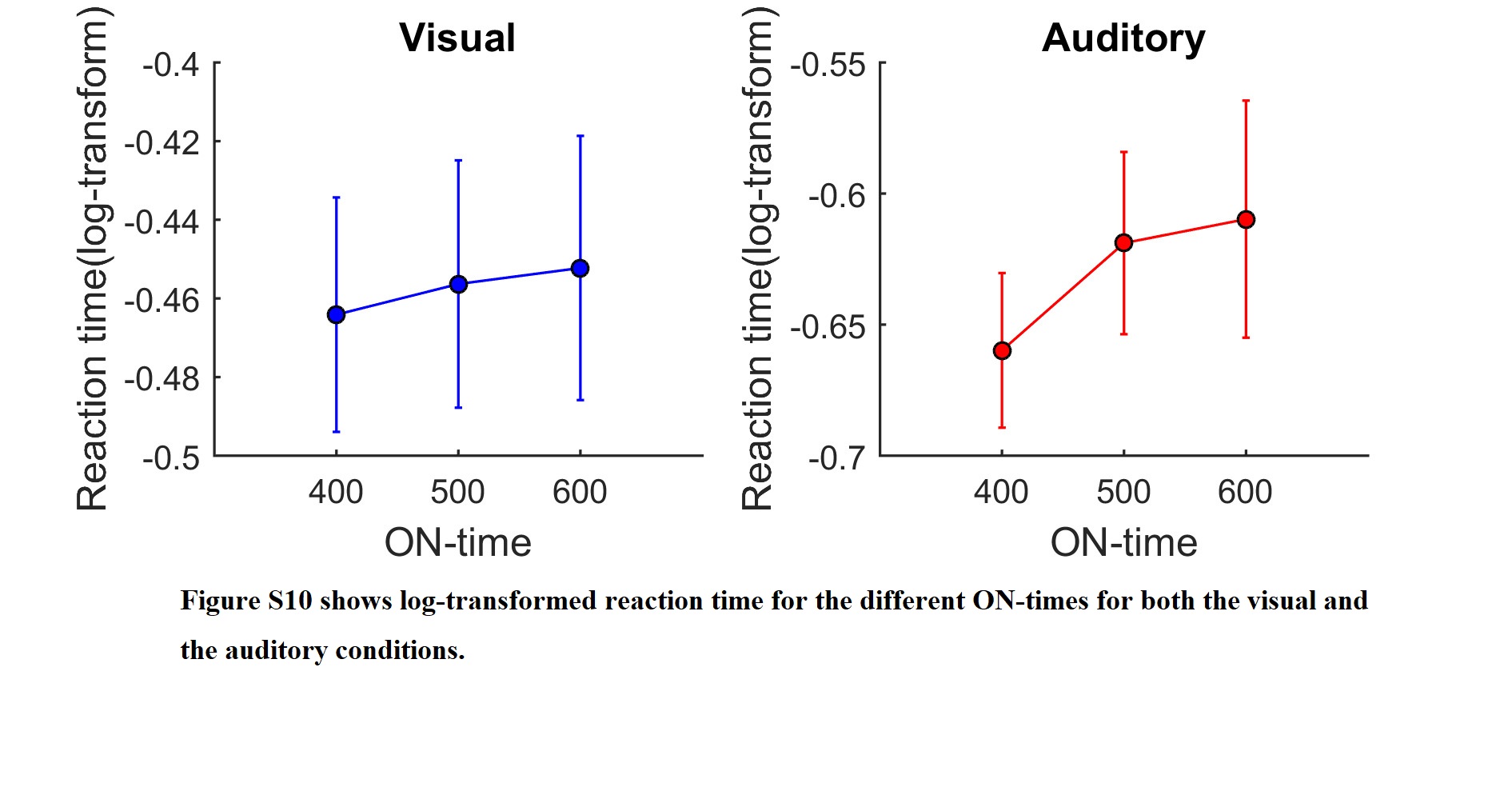

Supplement: Supplement 2 [file jovi-21-6-7_s002.jpg]

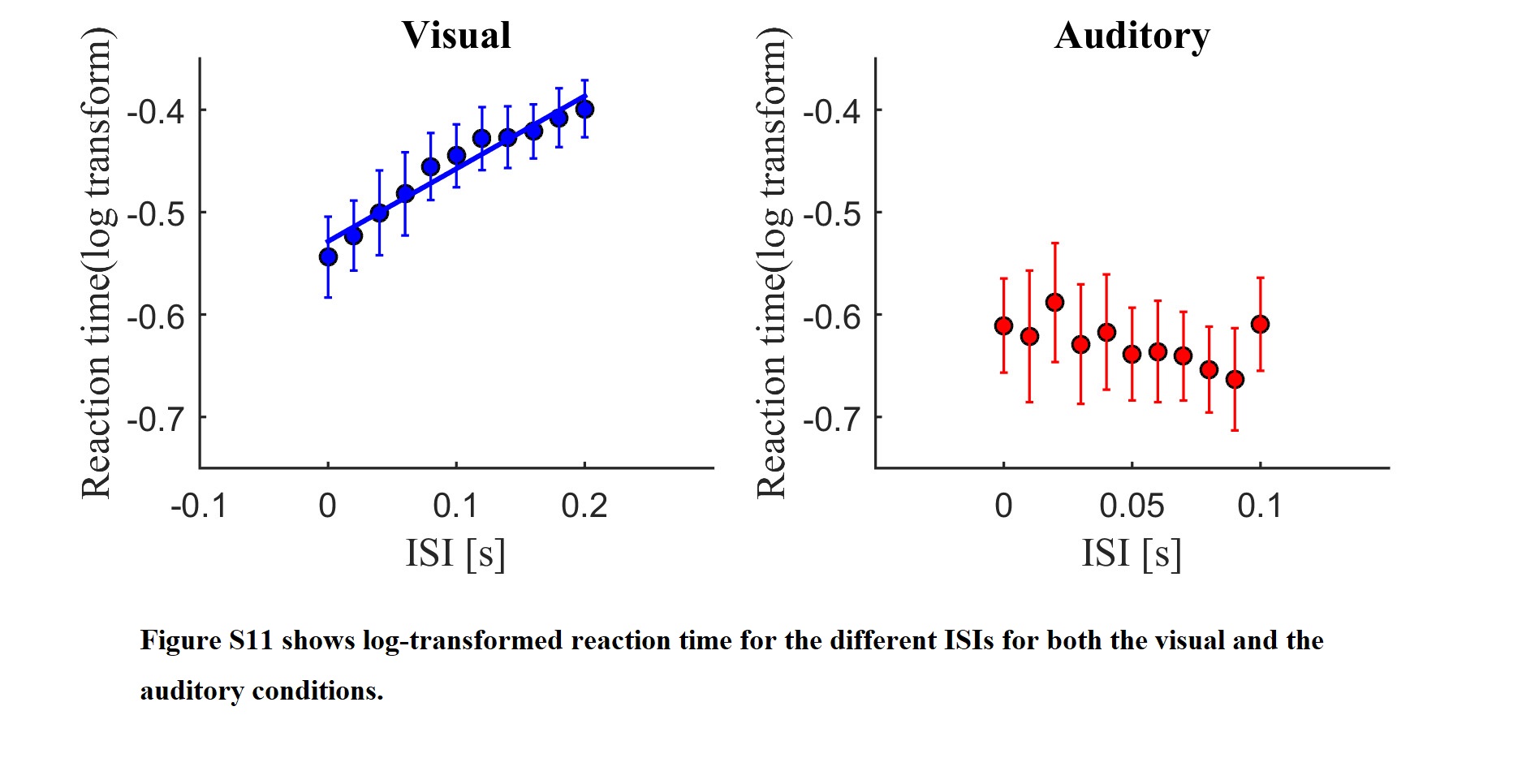

Supplement: Supplement 3 [file jovi-21-6-7_s003.jpg]

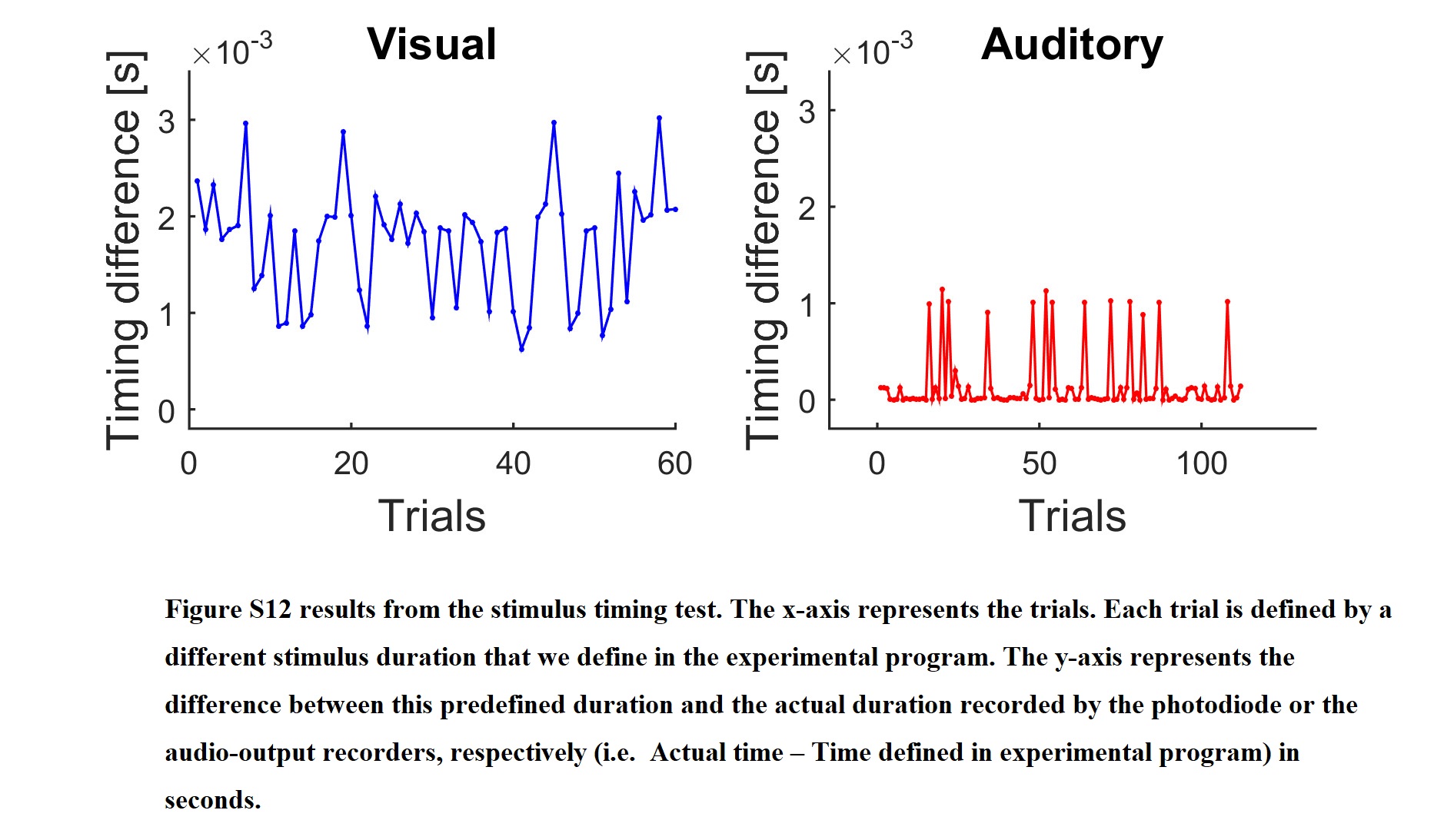

Supplement: Supplement 4 [file jovi-21-6-7_s004.jpg]

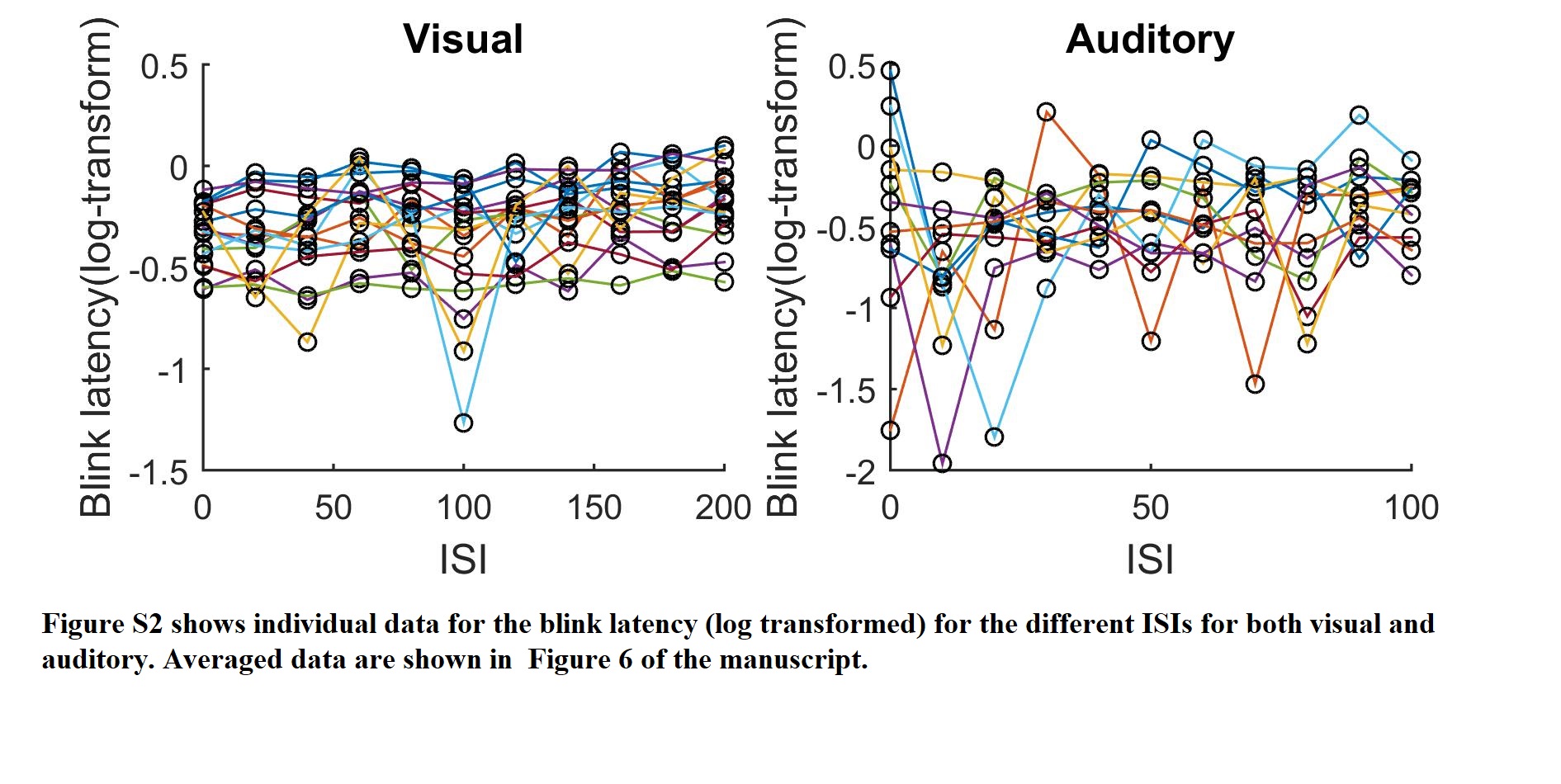

Supplement: Supplement 5 [file jovi-21-6-7_s005.jpeg]

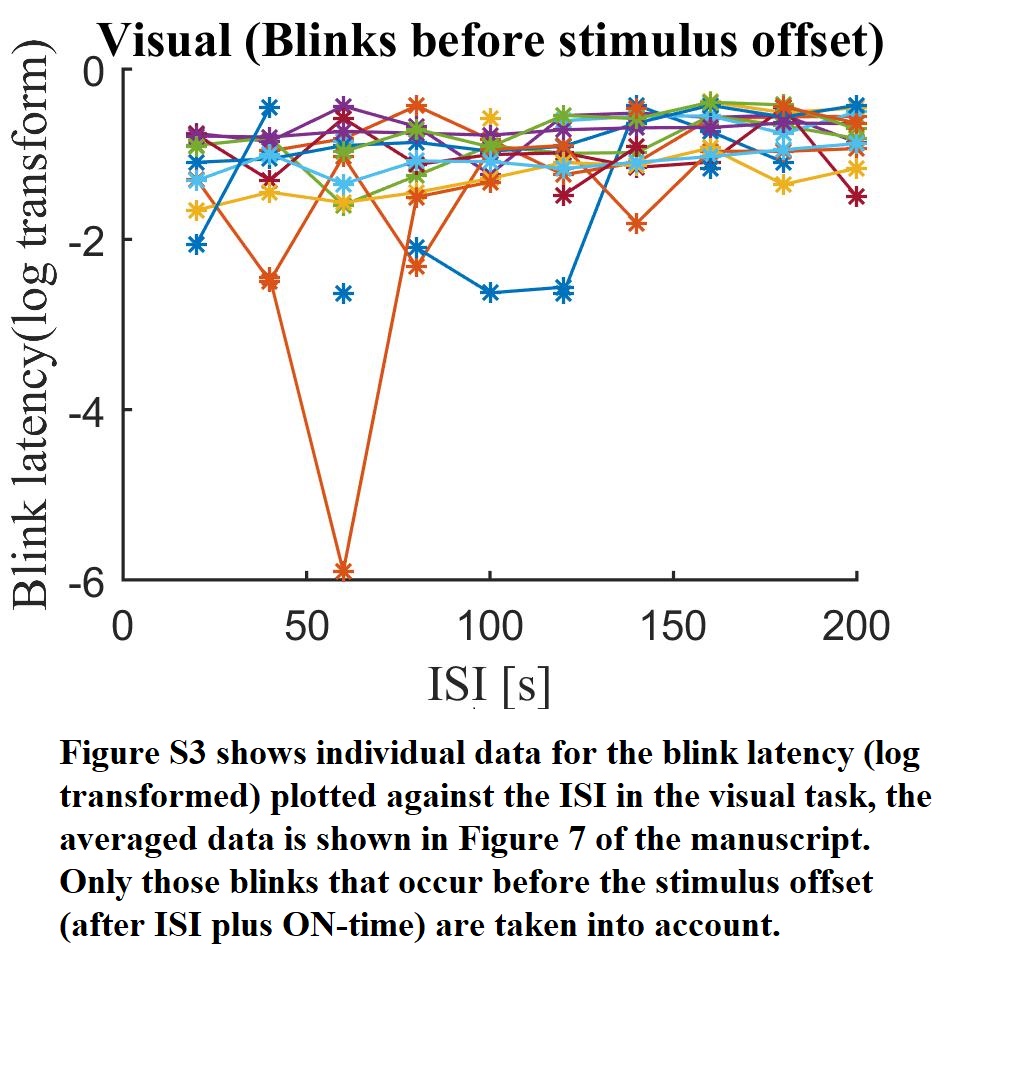

Supplement: Supplement 6 [file jovi-21-6-7_s006.jpeg]

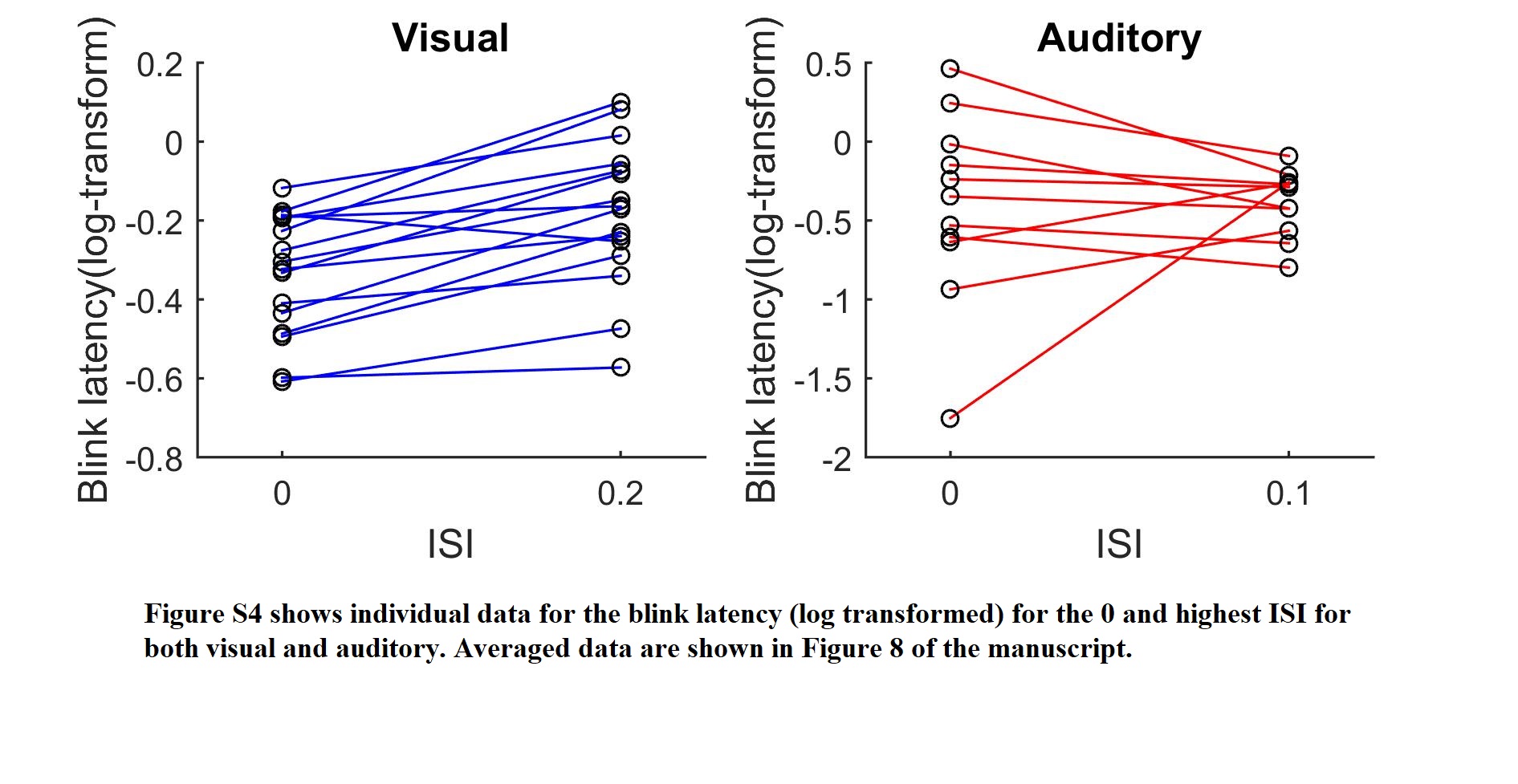

Supplement: Supplement 7 [file jovi-21-6-7_s007.jpeg]

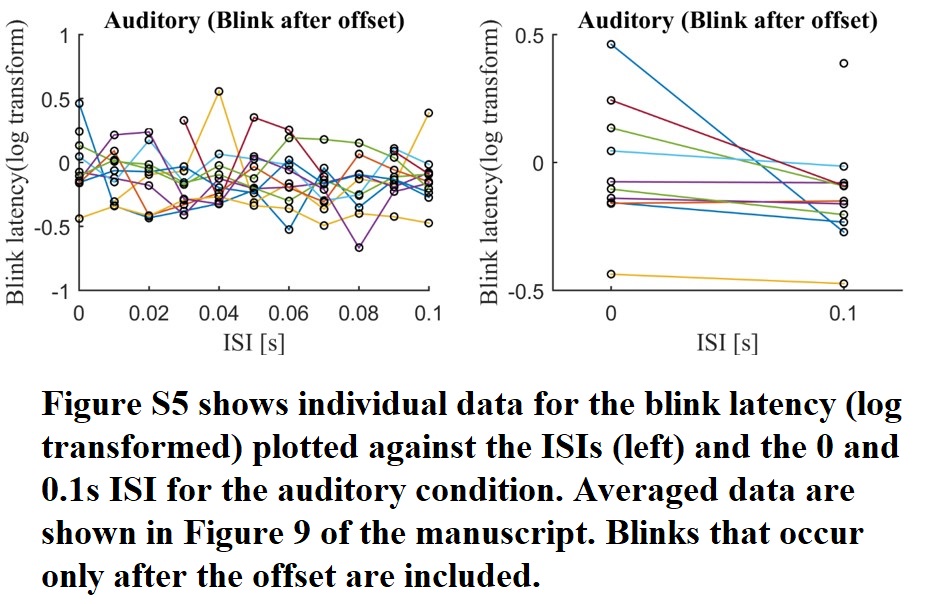

Supplement: Supplement 8 [file jovi-21-6-7_s008.jpg]

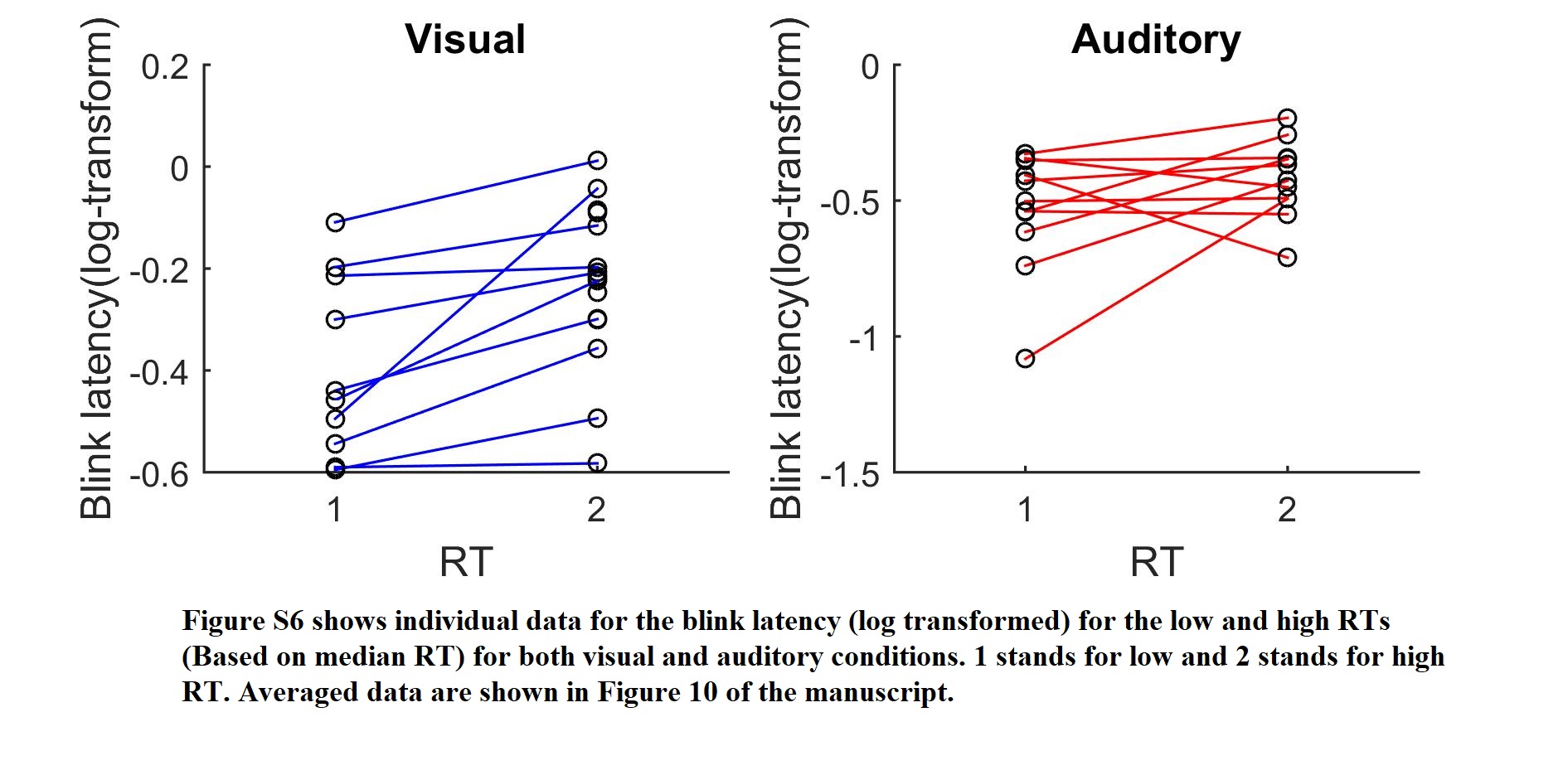

Supplement: Supplement 9 [file jovi-21-6-7_s009.jpeg]

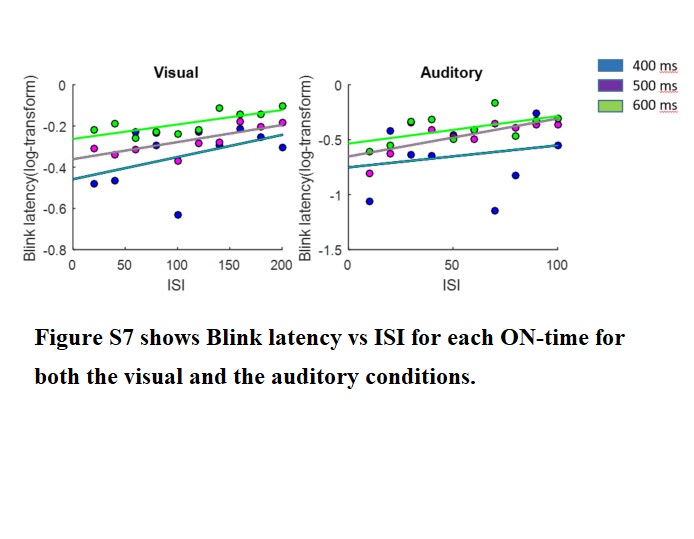

Supplement: Supplement 10 [file jovi-21-6-7_s010.jpg]

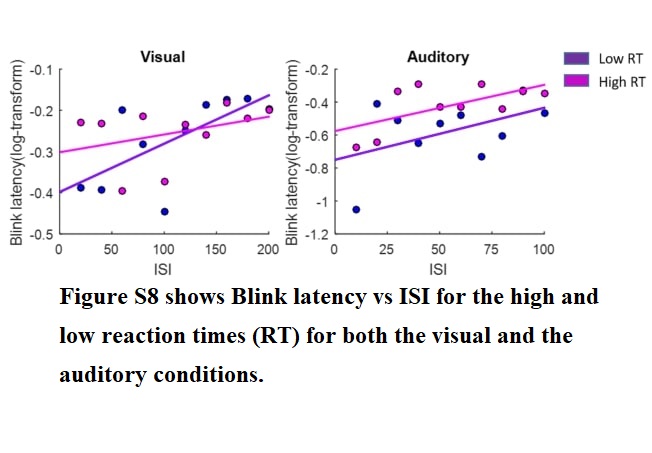

Supplement: Supplement 11 [file jovi-21-6-7_s011.jpg]

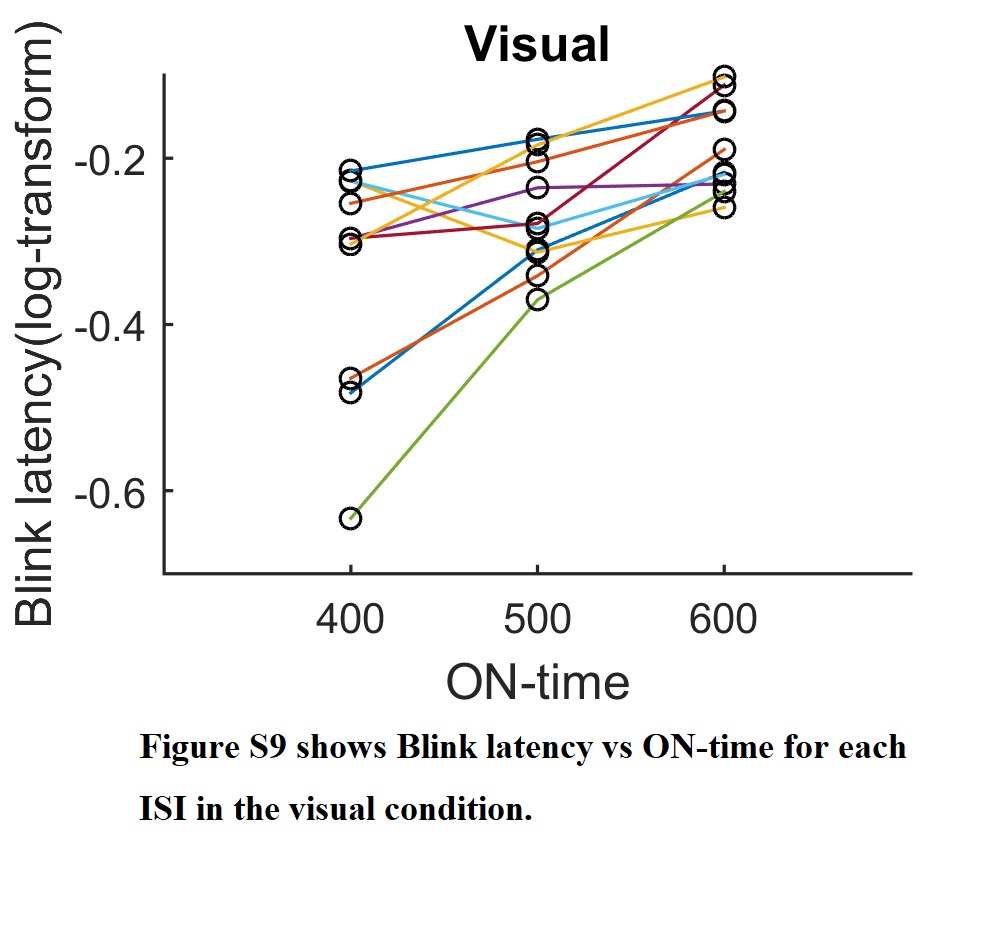

Supplement: Supplement 12 [file jovi-21-6-7_s012.jpg]
