# Supplementary material for: The latency of spontaneous eye blinks marks relevant visual and auditory information processing
Source: J Vis. 2021 Jun 11;21(6):7. doi: 10.1167/jov.21.6.7 (PMC8196427; doi:10.1167/jov.21.6.7)
Supplement: Supplement 13 [file jovi-21-6-7_s013.pdf]

Supplementary material

The Latency of Spontaneous Eye Blinks Marks Relevant Visual and Auditory Information Processing

Supriya Murali[1], Barbara Händel[1]

[1]Department of Psychology III, University of Würzburg

Section 1: Individual data

(Figure S1 to Figure S6)

Section 2: Blink latency for each ISI and ON-time, and for each ISI and RT combinations

(Figure S7 to Figure S9)

Section 3: Reaction time for the different ON-times and reaction times

(Figure S10 to Figure S11)

ANCOVA results:

1. For the visual, there is a significant effect of ISI ($F(1,527) = 26.8$, $p = .0001$) but not ON-time ($F(2,527) = 2.4$, $p = .09$) and no interaction ($F(2,527) = 0.92$, $p=.40$)

2. For the auditory there is no significant effect of ISI ($F(1,681) = 1.47$, $p = .25$), but significant effect of ON-time ($F(2,681) = 5.71$, $p = .004$) and no interaction ($F(2,681) = 2.8$, $p = .06$).

Section 4: Stimulus timing test results

(Figure S12)

The results of the test revealed that the mean difference (over the trials) between the experimental input and the actual recording for the visual was 0.0017s (SD = 5.9265e-04s) and the auditory was 1.5364e-04s (SD = 3.1789e-04). Both domains therefore show an temporal accuracy of below 2 ms including stimulus on- and offset.

Section 5: Blink latency vs reaction time for each subject

Table S1

Results from the individual regressions for blink latency vs response time for the visual condition. Note that two subjects had been excluded (see Methods).

Subject number	Beta	R^2	F	p
1	-0.25	0.01	1.2	0.26
2	-0.37	0.01	1.8	0.17
3	-	-	-	-
4	0.5	0.21	18.1	***6.59e-05****
5	-0.18	0.005	0.69	0.41
6	-0.75	0.06	6.4	***0.01****
7	-0.08	0.02	1.25	0.26
8	-0.02	0.0002	0.09	0.76
9	0.05	0.004	0.57	0.45
10	-0.08	0.006	0.404	0.53
11	0.04	9.25e-05	0.05	0.82
12	0.5	0.07	44.08	***7.3e-11****
13	0.2	0.01	0.46	0.5
14	0.03	0.0001	0.05	0.82
15	0.02	0.0004	0.02	0.873
16	-	-	-	-
17	-0.64	0.05	2.5	0.11
18	0.09	0.01	2.81	0.1

Table S2

Results from the individual regressions for blink latency vs response time for the auditory condition. Note that one subject had been excluded (see Methods).

**Subject number**	**Beta**	**R^2**	**F**	**p**
1	0.57	0.1	12.3	***0.001****
2	-0.2	0.001	0.13	0.71
3	-0.92	0.1	3.5	0.07
4	0.46	0.04	1.9	0.17
5	-0.07	0.03	3.14	0.08
6	-0.81	0.1	3.7	0.06
7	-0.56	0.04	2.1	0.1
8	--	-	-	-
9	-0.07	0.004	0.1	0.74
10	-0.15	0.01	0.76	0.38
11	-0.16	0.01	1.87	0.17
12	0.07	0.0002	0.07	0.77
13	-0.53	0.08	2.12	0.15
14	0.09	0.003	0.91	0.34
15	0.13	0.005	0.34	0.55
16	0.15	0.01	3.5	0.06
17	-0.005	1.5e-05	0.001	0.97
18	0.22	0.02	3.22	0.07
